# Photocatalytic Reduction of Carbon Dioxide to Methanol: Carbonaceous Materials, Kinetics, Industrial Feasibility, and Future Directions

**DOI:** 10.1021/acs.energyfuels.3c00714

**Published:** 2023-05-16

**Authors:** Parameswaram Ganji, Ramesh Kumar Chowdari, Blaž Likozar

**Affiliations:** Department of Catalysis and Chemical Reaction Engineering, National Institute of Chemistry, Hajdrihova ulica 19, Ljubljana 1001, Slovenia

## Abstract

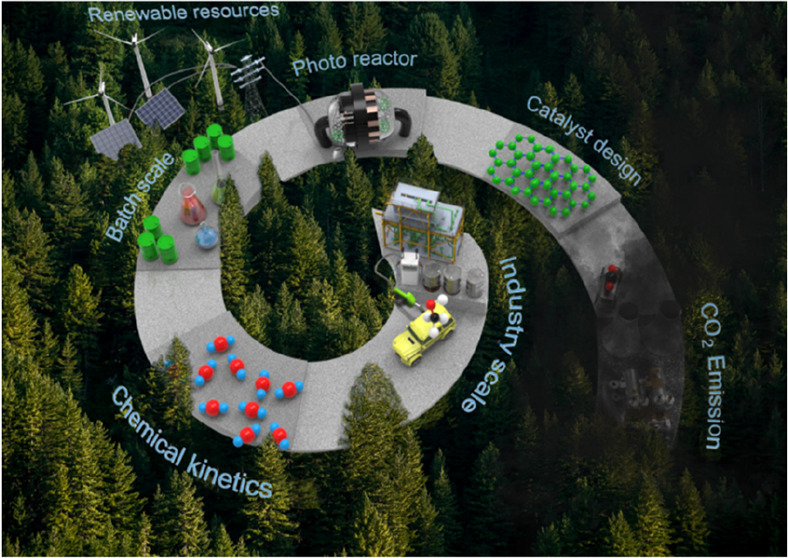

Photocatalytic carbon dioxide reduction (PCCR) for methanol synthesis (CH_3_OH) targeting renewable energy resources is an attractive way to create a sustainable environment and also balance the carbon-neutral series. The application of PCCR to methanol enables the generation of solar energy while reducing CO_2_, killing two birds with one stone in terms of energy and the environment. In recent years, research on CO_2_ utilization has focused on hydrogenation of CO_2_ to methanol due to global warming. This article mainly focuses on selective carbonaceous materials such as graphene, mesoporous carbon, and carbon nanotubes (CNTs) as catalysts for heterogeneous photocatalytic CO_2_ reduction to methanol. In addition, special emphasis will be placed on the state of the art of PCCR catalysts as this type of research will be of great benefit for further development in this field. The main features of the reaction kinetics, techno-economic study, and current technological developments in PCCR are covered in detail.

## 1 Introduction

Nowadays, one of the major challenges around the world is protecting the environment from various problems such as global warming, industrial effluents, wastewater treatment, etc. One of the biggest problems is global warming, which is caused by the uncontrolled emission of greenhouse gases into the environment, which is caused by the high use of fossil fuels for transportation. This is mainly due to the economy and growing population, the global challenges of modern society, etc.^1,2^ In order to protect the environment, many countries around the world have enacted strict environmental laws. For example, in the EU, policies such as the European Green Deal, Fit for 55, UN Sustainable Development Goals, etc., have been announced. Among the above-mentioned EU policies on European targets, most of them are achieved by reducing global warming to provide an environmentally safe, healthy, and quality life. Among all greenhouse gases, carbon dioxide is one of the most important, most emitted, and so far the main responsible gas. The calculated value of CO_2_ concentration in the environment is increasing worldwide and is reported to be 381 ppm for the year 2006. By 2020, the value is expected to reach 413 ppm by 2020.^3^ For this reason, the global scientific community, universities, and industry are promoting the use of CO_2_ to produce valuable chemicals and/or fuels, which not only helps to curb global warming but also creates an environmentally friendly atmosphere. Several effective pathways for CO_2_ conversion have been reported in the literature, such as organic synthesis, thermocatalytic, electrocatalytic, and photocatalytic processes.^4−12^ These pathways produce the following value-added chemicals: urea, syn gas, methane, formic acid, formic acid derivatives, carbonates, oxygenates (methanol, dimethyl ether, and ethanol), and hydrocarbons.^13−16^ Based on available data, technology transfer in converting CO_2_ into valuable chemicals from laboratory/microscale to market scale has been limited to date. Among all CO_2_ hydrogenation products, methanol is one of the most interesting and promising chemicals because it has a high density like a solar liquid fuel, and electricity can be obtained in a single step by direct methanol fuel cells. This is the main reason most researchers are focusing on CO_2_ valorization recently. Thus, methanol production is expanding as it is used as an alternative fuel and valuable chemical,^14,17−19^ so the large-scale application of this process is of great importance. Another advantage is that methanol can serve as a feedstock for a wide range of synthetic chemicals. In addition, methanol is commercially used in fuels to blend with gasoline and increase the octane rating.^20^ Therefore, methanol is one of the most important chemical feedstocks that has a major impact on the global economy. Globally, methanol consumption accounts for 40% of total energy. In 1994, George Olah planned to transform the fossil hydrocarbon system into a ‘methanol economy’ in which CH_3_OH, obtained by reducing CO_2_, would be used as a feedstock for energy storage and also for transportation because it has a very good energy yield per unit mass, 20.1 MJ/kg. The standard reduction potential (SHE at pH = 7 and 25 °C) for CO_2_ conversion is −0.38 V and is as follows:
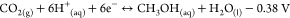
1

For the use and conversion of CO_2_ into valuable products, it is important to know the global emissions, the main sources, the different strategies to convert CO_2_, etc. In the following section, you will find a brief description of CO_2_ emission and the different conversion pathways.

### CO_2_ Emission, Utilization, and Conversion Routes

Available data from previous reviews reports^21,22^ indicate that China is the largest polluter, and ranking first in CO_2_ emissions, at ∼9.90 GT per year or nearly 29% of the global total. The next largest polluter is the United States with 4.70 GT/year, accounting for 14% of the world, and other countries such as India have a share of only 2.30 GT (2019). Global CO_2_ emissions depend on the policies and industrial development of each country. Therefore, the impact of CO_2_ emissions varies depending on each country’s environmental legislation and industrialization capacity, as well as population development.^23,24^ According to annual data, CO_2_ emissions increased gradually from 2010 to 2017. After that, very high CO_2_ emissions were observed, i.e., ∼34,000 million tons in 2018–2019,^25^ which could be due to the soft GDP growth, and the increase in energy prices also did not reduce consumption but increased it every year.^26^ In the recent review of 2020, CO_2_ emissions were reported to reach 35.2 BMT.^25^ Therefore, it is necessary to develop scientific methods to convert, i.e., capture, and then reuse the CO_2_ emissions generated from the use of fossil fuels (coal, natural gas, etc.) to produce useful chemicals.

**Figure 1:**
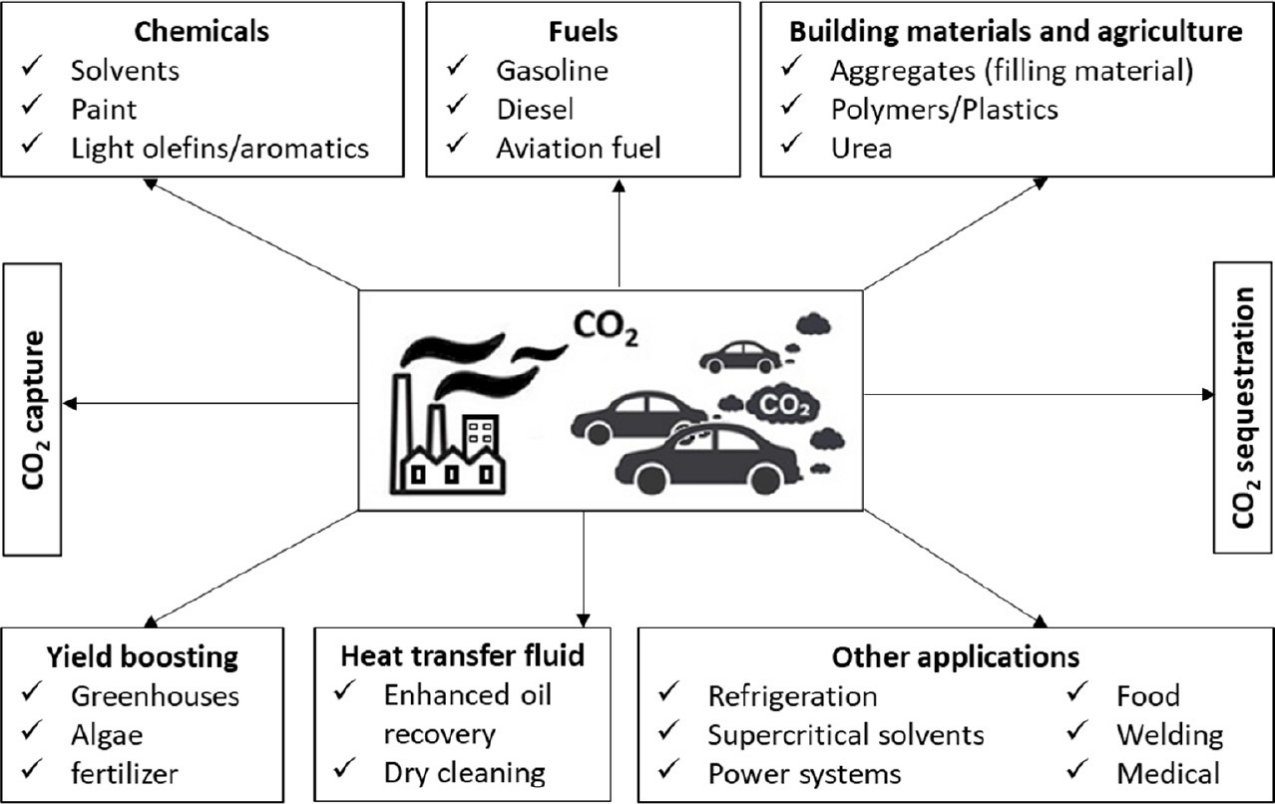
Simple classification of CO_2_ utilization pathways.^30^ Reproduced with permission from ref (30) (Redrawn). Copyright 2021, Frontiers Media S.A.

The use of CO_2_ is a favorable way to decline the global warming, and another interesting point is the escalation of fossil fuels replacement. Another route to reduce CO_2_ emissions is through CCS, a technology also described in the literature.^19^ However, the CCS technology is very expensive and not economically viable because the process is very energy intensive, and the main drawback of CCS is the escape of CO_2_ from the stored material. Figure 1 shows different CO_2_ utilization pathways such as nonconversion, catalytic conversion, biological conversion, etc. As a raw material, CO_2_ utilization will create a potential market value for the products or services that use these methods.^27,28^ Currently, profitable industries that use CO_2_ include food and beverage processing, metal fabrication, petroleum refining, and firefighting as a flame retardant. In recent years, more attention has been paid to the chemical and biological uses of CO_2_ for fuels,^29^ chemicals, and building materials. However, the implementation of these processes into commercial practice is still ongoing.

**Figure 2:**
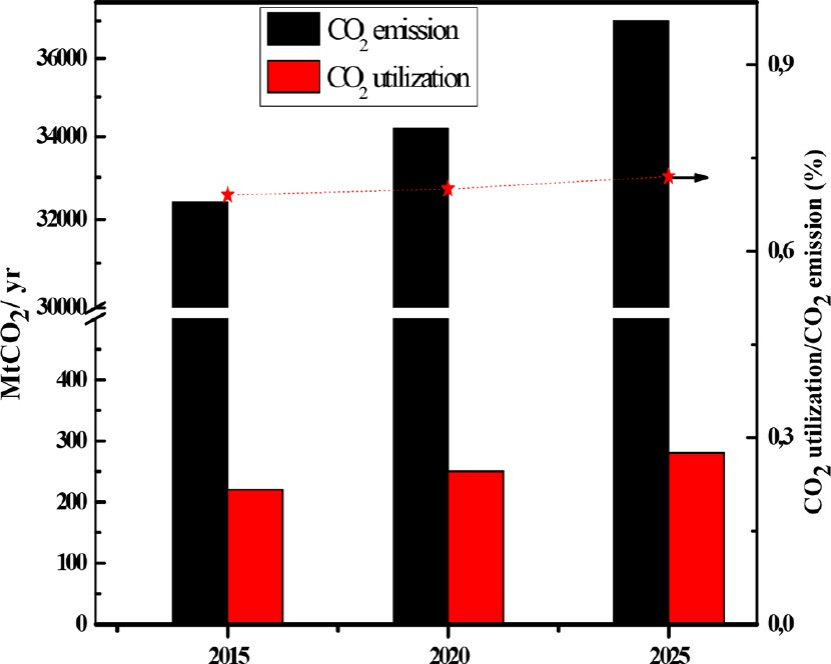
Global emission and utilization of CO_2_. Note: Projections for future global CO_2_ demand are based on an average year-on-year growth rate of 1.7% (International Energy Agency, 2019). Projections for future global CO_2_ emission are based on an average year-on-year growth rate of 1.4% (based on the annual average growth rate of 2009–2019).^26,30^ Reproduced with permission from ref (30) (Redrawn). Copyright 2021 Frontiers Media S.A.

Figure 2 shows that the ratio of CO_2_ use to CO_2_ emissions is <1%, suggested by Dudley projections. Over the period 2015–2025, only a slight increase in CO_2_ use is observed, while there are drastic fluctuations in CO_2_ emissions. The largest consuming sector is agriculture (130 Mt/yr CO_2_) in the form of urea production, and the next largest sector is the oil industry (70 to 80 Mt/y CO_2_), where CO_2_ is used for EOR.^31^ Currently, two-thirds of the global demand for CO_2_ use is observed in North America (33%), China (21%), and Europe (16%), so the demand for CO_2_ use is increasing year by year.^26^ As mentioned earlier, the conversion of CO_2_ into valuable chemicals is limited by market size. For this reason, the goal is to develop an efficient process to convert CO_2_ into methanol, which could be used as fuel and valuable chemicals.^14,17−19^ Commercial markets have recognized that the main source of CO_2_ is fossil fuels, so a CO_2_ levy in modern workplaces can give CO_2_ utilization a business appeal. Some start-up companies, such as OPUS12, CERT, Dioxide Materials, and established companies, such as Siemens, are also working on CO_2_ reduction techniques as a first step toward a large-scale process. There are examples of functioning industries for CO_2_ utilization in many countries, such as Iceland (George Olah production plant), the USA (Century production plant), Canada (Weyburn-Midale CO_2_ project), China (China National Petroleum Company, CNPC, Jilin Oil Field CO_2_ EOR), Norway (Sleipner CO_2_ Storage), and Western Australia (Gorgon CO_2_ Injection), etc., and many more industries are being planned. Therefore, the global utilization of CO_2_ is currently an important area that needs to create a sustainable environment.

**Figure 3:**
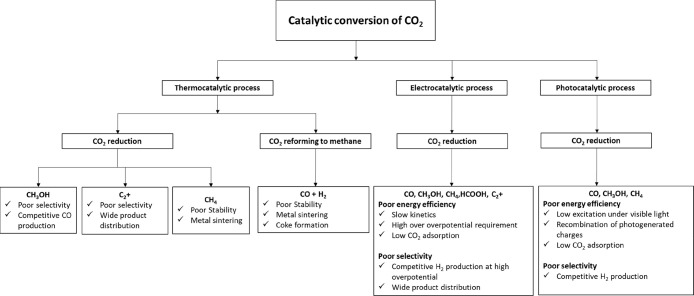
Catalytic conversion of CO_2_*via* different routes.^32^ Reproduced with permission from ref (32). Copyright 2020 Royal Society of Chemistry.

Conventionally, the conversion of CO_2_ has been carried out by thermocatalysis,^21,32^ electrocatalysis,^27,33^ and photocatalysis^34−36^ to obtain methanol as a product. Therefore, in the following section, we briefly review the different catalytic systems used for the reduction of CO_2_ by the main routes. Since our area of interest is the photocatalytic approach to CO_2_ reduction, we focus on carbonaceous materials, since there are fewer publications and review articles on this than on the general type of catalysts already published, as well as several review articles on this topic.^25,30,37^Figure 3 shows the main catalytic routes for the conversion of CO_2_ into valuable products. A brief description of the thermo- and electrocatalytic conversion of CO_2_ is provided below.

### Thermocatalytic Conversion of CO_2_:

Catalytic thermal reduction of CO_2_ to methanol is one of the attractive approaches and is already described in numerous literature reports.^20,21,30,38^ The use of CO_2_ to produce methanol by catalytic hydrogenation of CO_2_ is a possible solution for CO_2_ capture and energy storage. In this process, we obtain methanol with a neutral carbon footprint, which can be used as an unpolluted energy source and produces less particulate matter and no/low NO_*x*_ (nitrogen oxides). The production of methanol by thermocatalytic conversion of CO_2_ is already used industrially. In 1923, methanol was produced on an industrial scale by converting coal to syn gas. We are all very grateful to Alwin Mittasch and Mathias Pier of BASF for this work. Then, in the 1940s, the process for producing methanol from synthesis gas was introduced,^39^ which was used worldwide in the 1960s.^40^ Since the 19th century, the greatest achievement has been methanol production, and there are currently more than 90 plants in operation worldwide. About 2,00,000 tons of CH_3_OH are used daily as a chemical feedstock or as a fuel in the transportation sector. Several research groups have reported a variety of heterogeneous catalysts, e.g., catalysts based on Cu, Zn, Au, Ag, Cr, and Pd metals, In_2_O_3_, oxygen-deficient ZnO-ZrO_2_ materials, etc., for methanol production from CO_2_ hydrogenation.^32,41−43^ In the literature, most studies from industry and academia refer to copper-based (Cu) metal catalysts and also to commercially available Cu-ZnO-Al_2_O_3_ for the selective formation of methanol from the hydrogenation of CO_2_. Some of the selected literature reports on catalyst systems for the hydrogenation of CO_2_ to methanol are listed in Table 1. All of the catalysts described in this section are effective; however, further studies are needed before we can conclude on the practicality of such catalysts in terms of yield, selectivity, and long-lasting activity.

**Table 1:** Selected Catalytic Systems for the Methanol Synthesis from Hydrogenation of CO_2_

S. No.	catalyst	methanol space time yield	year	ref.
1	Cu/MgO-TiO_2_	56%	2016	(44)
2	Pd–Cu/SiO_2_	1.12 STY.mol^-1^.kg_cat_^–1^.h^–1^	2015	(42)
3	Cu-ZnO-Al_2_O_3_	0.15 g. mL^–1^.h^–1^	2013	(45)
4	In_2_O_3_/ZrO_2_	9.22 STYmol.^-1^kg _cat_^–1^ h^–1^	2016	(46)
5	CuIn@SiO_2_	0.21 g. g _cat_^–1^.h^–1^	2019	(47)
6	Pd/ZnO@ZIF-8	0.46 g. g_cat_^–1^.h^–1^	2019	(48)
7	8 wt % Cu/ZnAl_2_O_4_	242 g_CH_3_OH_.kg_cat_^–1^.h^–1^	2023	(49)
8	In_80_Ce_20_	3.27 g_CH_3_OH_·m_cat_^–2^·h^–1^	2023	(50)

### Electrocatalytic Conversion of CO_2_

The electrochemical carbon dioxide reduction (ECDRR) process can be used as an inspiring technique for CO_2_ reuse and storage from a financial and environmental point of view. In the ECDRR process, the conversion of CO_2_ to selective methanol formation relies on electrocatalysts, as they play a key role in the overall reaction. ECDRR experiments have been performed since the 1950s, but the scientific community still faces several challenges because the functions for high catalytic activity, selectivity, and other factors are not clear.^51^ In 1985, Hori et al.^52^ first reported a comprehensive electrochemical study in which they found ECDRR products in liquid and gaseous forms and quantified them with significant Faradaic efficiency (FE = 100%).

Electrochemical activation of carbon dioxide with electrocatalytic materials yields methanol from the hydrogenation of CO_2_ under mild reaction conditions. Some of the noble metal catalysts (Pt, Pd, and Ru)^53−55^ are being investigated for electrochemical CO_2_ reduction, along with Na- or K-modified β-alumina as a support for the chemisorption of carbon dioxide and hydrogen on the active metal surface.^54,55^ Low-cost metals, e.g., copper (Cu) on K-β-Al_2_O_3_^56^ or nickel (Ni) on Y_2_O_3_-stabilized ZrO_2_(YSZ),^57^ etc., are also being considered for the electrochemical conversion of CO_2_. Table 2 shows the reported electrocatalytic systems for the conversion of CO_2_ to methanol.

**Table 2:** Selected Catalytic Systems for the Methanol Synthesis from Electrocatalysis of CO_2_

S. No.	catalyst	methanol product (FE,%)	potential (vs RHE, V)	electrolyte	year	ref.
1	Cu/carbon 1000	3	–0.7	0.1 M KHCO_3_	2017	(62)
2	30% Cu_2_O-MWCNTs	38	–0.8	0.5 M NaHCO_3_	2016	(33)
3	FeP nanoarray	80.2	–0.20	0.5 M KHCO_3_	2020	(63)
4	Mo–Bi BMC	71.2	–0.7	0.5 M [Bmim]BF_4_	2016	(64)
5	Pt_*x*_Zn/C (1 < *x* < 3)	81.4	–0.90	0.1 M NaHCO_3_	2020	(65)
6	C-Py-Sn-Zn	59.9	–0.5	KHCO_3_	2020	(66)
7	Sn/CuO	88.6	0.35	[Bmim]BF_4_/ H_2_O	2021	(67)
8	Cu-*g*-C_3_N_4_/MoS_2_	19.7	–1.4	**-**	2022	(68)

In 2020, our research group^27^ and other research groups from around the world presented reviews on ECDRR,^58−60^ in which the general discussion referred to nanostructured single-atom catalysts (SACs) based on gold (Au), silver (Ag), and copper (Cu), such as Ni-SACs, Mn-SACs, Fe-SACs, Co-SACs, etc. Nevertheless, the scientific community is in search of a novel metal-based nanostructure catalyst that can provide a high yield of methanol with high catalyst stability. Again, we have found some of the most comprehensive literature reviews on electrochemical approaches to CO_2_ reduction available to interested readers in ECDRR.^25,61^

### Photocatalytic Conversion of CO_2_

PCCR is a method that “kills two birds with one stone” to save energy while protecting our environment from global warming. Inoue and his collaborators pioneered the photocatalytic conversion of CO_2_ to hydrocarbons in 1979.^69^ This is the first demonstration in which they used TiO_2_, CdS, and GaP materials for the conversion of CO_2_. Later, nanocomposites of TiO_2_, and nonmetallic catalysts were investigated for PCCR with H_2_O and light irradiation.^70−74^ Photocatalysts such as metal complexes,^75,76^ semiconductor nanomaterials,^77−79^ and photoelectrodes^80,81^ were also investigated for PCCR systems. The factors limiting the productivity of artificial photosynthesis are the rapid recombination of charge carriers, the mismatch between the band gap of the photocatalyst and the spectrum of solar radiation, and the unfavorable position of the band-edge.^6,7,82^ Therefore, the practical importance of this area of PCCR research has led to an inordinate interest in the development of active photocatalysts and reaction assemblies.

**Figure 4:**
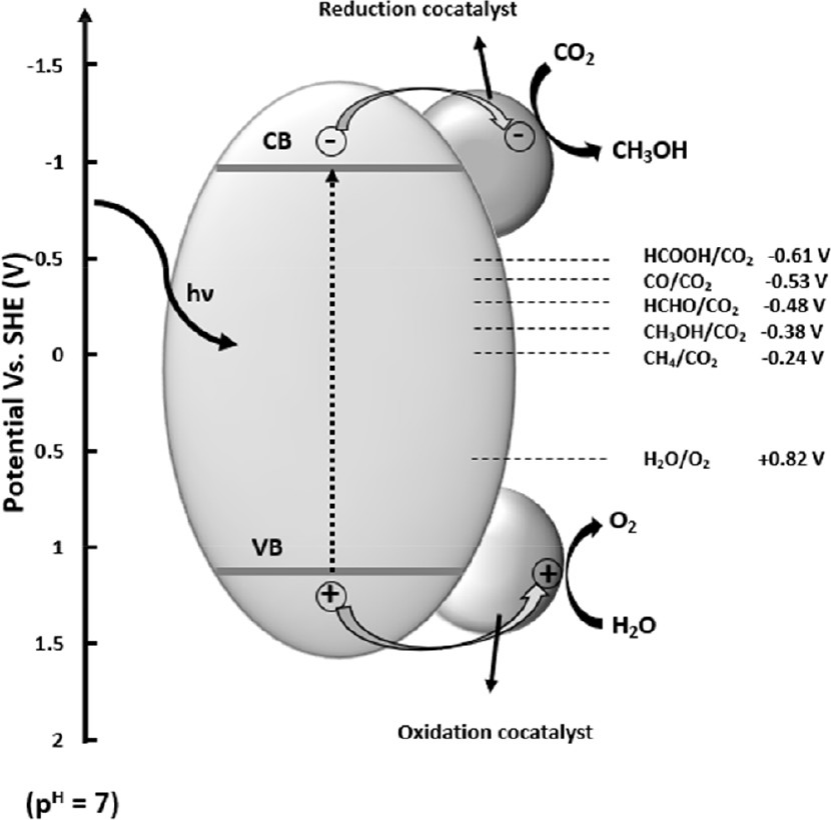
Schematic diagram of the photocatalytic conversion of CO_2_.^93^ Reproduced with permission from ref (93). Copyright 2018 Wiley-VCH Verlag GmbH & Co. KGaA, Weinheim.

When we compare photocatalysis with thermocatalytic processes, the general question arises: “Why cannot PCCR compete with the conventional CO_2_ hydrogenation reaction in terms of activity and selectivity?” The main reason is that the C–O bond can be easily cleaved in the thermocatalytic process because high temperature and pressure are applied. On the other hand, the activity in PCCR and other systems is more complex and has never been achieved before. In the mechanism of PCCR system, three different phases are observed: (1) absorption of incident photons, (2) generation, separation, and transfer of electron–hole pairs (e^–^–h^+^), and (3) adsorption, activation, and conversion of CO_2_ molecules on the surface.^83^Figure 4 shows a general schematic representation of the photocatalytic reduction of CO_2_. In this process, the photoradiation from the catalysts causes electron transfer from VB to CB, reducing the CO_2_ molecule and forming CH_3_OH (see eq 1; Figure 4). Subsequently, researchers focused on water splitting by photocatalysis because the initial research mainly focused on light collection, charge separation, and charge energy level.^84−86^ These points are very similar to the performance of semiconductors, so this issue is more important for the efficiency of photocatalytic reactions, such as water splitting. Based on the photocatalytic mechanism for CO_2_ reduction to methanol, several catalytic systems have been described in the literature, such as one-dimensional TiO_2_ single crystals coated with ultrafine Pt NPs,^87^ 1.2% Cu-TiO_2_,^88^ 2% Cu-TiO_2_,^35^ 0.5% Au on TiO_2_ NWs,^89^ 3% Cu-TiO_2_ (Evonik P-25),^90^ N-TiO_2_ NTs,^91^ graphene/N-TiO_2_,^92^ etc.

In summary, all three described processes for CO_2_ reduction to methanol are based on metals/metal oxides and semiconductor materials. Of all these processes, photocatalytic conversion is of increasing interest, as this route is practical, sustainable, and environmentally friendly. However, as mentioned above, this photocatalytic conversion process requires great attention in the preparation of an effective catalyst to obtain a high yield of methanol and a sustainable atmosphere. In this context, we have focused on a new generation of carbonaceous materials for the photocatalytic conversion of CO_2_ to gain a deeper understanding of visible light absorption, structural surface properties, charge carrier acceptance, reactant adsorption, product desorption, etc., all of which need to be carefully and comprehensively studied. This is because reports on carbonaceous materials are scarce and not very concentrated. Therefore, in this review article, we mainly focused on the application of carbonaceous materials for methanol production from carbon dioxide. In particular, the role of carbonaceous materials in composite materials for photocatalytic reduction of CO_2_ is discussed. This review also includes the kinetics of CO_2_ reduction at different scales (micro, meso, macro) and a discussion of techno-economic feasibility.

### Scope of the Review

In the last three decades, numerous research efforts have been made to develop active heterogeneous catalysts for the conversion of CO_2_ by three processes to produce methanol. A large number of review articles, publications, and books^27,30,37,94−97^ have been published on research topics of CO_2_ conversion such as CO_2_ utilization by thermal catalysis,^20,98^ electrocatalytic reduction,^99,100^ photocatalytic synthesis,^11,75,101−113^ plasma,^114^ and other methods.^115−117^ Recently, the research group of Shanmuga and Ramyashree published a review article on MOF compounds for PCCR of methanol.^118^ In 2022, Gawande’s group discussed recent developments in the photocatalytic hydrogenation of CO_2_ to methanol. In particular, the article described MOFs, mixed metal oxides, carbon, TiO_2_, and plasmon-based nanomaterials.^37^ Very few research articles have been published in the literature on carbon-based catalysts for the PCCR of methanol. In this review, attention is drawn to the PCCR of methanol with carbon-based materials, highlighting key features such as activity, product selectivity, catalyst reusability, etc. Finally, an overview of the current development and main challenges related to photocatalytic materials as well as future perspectives will be provided. Our goal is not to duplicate published concepts on PCCR but to summarize potentially unique active catalysts, structure–activity relationships, kinetic studies, and industrial feasibility studies. Based on this, we can understand the concerns about PCCR to methanol, scale-up from small, the importance of methanol in the future energy system, and technical feasibility. Consequently, further studies on the techno-economic evaluation of CO_2_ reuse tools, operation mechanisms, and related plans are also discussed, which are essential. We believe that this comprehensive review work will be a useful resource to guide researchers toward new state-of-the-art graphene-based solid catalysts and the application of nanoscale, nanostructured, and porous materials for potential methanol production from CO_2_. Moreover, PCCR of CO_2_ into methanol using carbonaceous catalysts is one of the most interesting strategies to reduce global warming, which is why we focused on it.

## 2 Carbonaceous Photocatalytic Materials for CO_2_ Reduction

**Figure 5:**
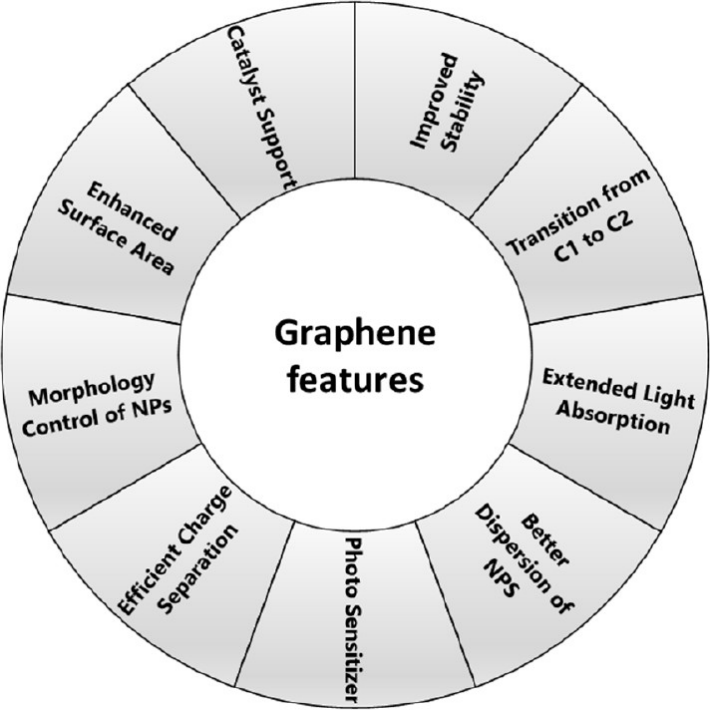
Features endowed by graphene/graphene derivatives to photocatalysts for improved activity of PCCR.^126^ Reproduced with permission from ref (126). Copyright 2019 Elsevier Inc.

As mentioned above, the main objective of this review is to discuss the photocatalytic reduction of methanol using carbonaceous catalysts. In this context, graphene is the most studied and reported material. Graphene consists of SP^2^ carbon with a single-layered, two-dimensional nanosheet with a hexagonally occupied lattice structure. Since the breakthrough discovery of graphene by Geim and Novoselov,^119−121^ it has been used in scientific and engineering fields. Graphene is also known as a wonder material due to its versatile properties, such as its large surface area, good conductivity, stability, and high flexibility.^122,123^ For this reason, graphene-based compounds are used as photocatalysts to improve PCCR efficiency. Graphene is used as platform chemicals and support materials from the chemical point of view, but it also improves the mechanical, catalytic, electrochemical, or photochemical properties. Moreover, the great advantage of graphene is that it can be easily obtained by the Hummers method using inexpensive graphite.^124,125^ For photocatalytic applications, TiO_2_ may be a suitable candidate for charge transfer to graphene. Therefore, the addition of graphene to a photocatalyst brings fabulous properties, some of which are summarized in Figure 5.

Another form of carbon allotrope is the CNT, first described by the great scientist Ijima in 1991. To produce MWCNTs, the arc discharge method is applied to carbon black.^127^ MWCNTs look like tubes of graphite with at least two or more layers, and their outer diameter is in the range 3–30 nm. MWCNTs are used as supports in many applications, such as CO and CO_2_ hydrogenation reactions, due to their excellent properties such as (i) their high aspect ratio (length/diameter), (ii) specific surface area, (iii) mechanical strength, (iv) rigidity, (v) electrical conductivity, (vi) thermostability, and (vii) tunable surface chemistry.^128−132^ Researchers found that Cu-ZnO-Al_2_O_3_ and Co–Cu catalysts with CNTs exhibited high activity and selectivity of alcohol in the CO/CO_2_ hydrogenation reaction.^128,129^ In addition, the group of Zhang et al. succeeded in synthesizing a highly active catalyst, i.e., Pd-ZnO/CNTs, for the hydrogenation of CO_2_ to CH_3_OH.^130^

Mesoporous carbon is another carbonaceous material for PCCR to methanol, and this capable material also has several applications as this material is used as support and in energy storage devices. The highly OMC material with its structural composition was first described by Ryoo et al.^133^ using mesoporous silica as a template. Subsequently, this OMC has been prepared by various synthetic methods for a variety of applications.^134,135^ In the following section, a detailed part of the application of carbonaceous materials for PCCR to CH_3_OH is discussed.

### 2.1 Role of Carbonaceous Material in Photocatalysis

Carbonaceous materials, especially graphene, are known for their excellent electron transport properties, which greatly improve the efficiency of catalytic electron–hole separation. This is the main advantage of graphene, and therefore, it is often used in PCCR to enhance the activity of the material. Graphene alone cannot be used as semiconductor because it has no band gap. In semiconductor–graphene hybrids, the extended conjugation of carbon in graphene sheets facilitates better charge transport on their surface, which enables efficient charge separation. The introduction of defects into the lattice of graphene sheets by doping with heteroatoms or oxidation can transform conductive graphene into a semiconductor. Moreover, the band gap can be tuned by systematic approaches.^119^ For example, to improve the photocatalytic efficiency, the WSe_2_ nanocomposite is combined with graphene nanosheets because graphene is an electron acceptor/transporter that plays an important role in separating the transport electron–hole pairs in the binary system.^121,123^ This is due to the ultrahigh electron conductivity of graphene, which enables the flow of electrons from the semiconductor to its surface, resulting in efficient electron–hole separation. In addition, the potential of graphene/graphene^–^ (−0.08 V vs standard hydrogen electrode (SHE), pH = 0) is typically lower than the conduction band potential of the photocatalyst, allowing for rapid electron migration from the photocatalyst to the graphene. The average lifetime of the electron–hole pairs is in the range ∼10^–7^–10^–5^ s. Thus, the dual role of graphene in the composite is enhanced by (1) the separation of electron–hole pairs is enhanced by injecting electrons from the conduction band of the photocatalyst (e.g., TiO_2_) into the graphene and (2) the recombination of electron–hole pairs in the excited photocatalyst (TiO_2_) is greatly delayed.

**Figure 6:**
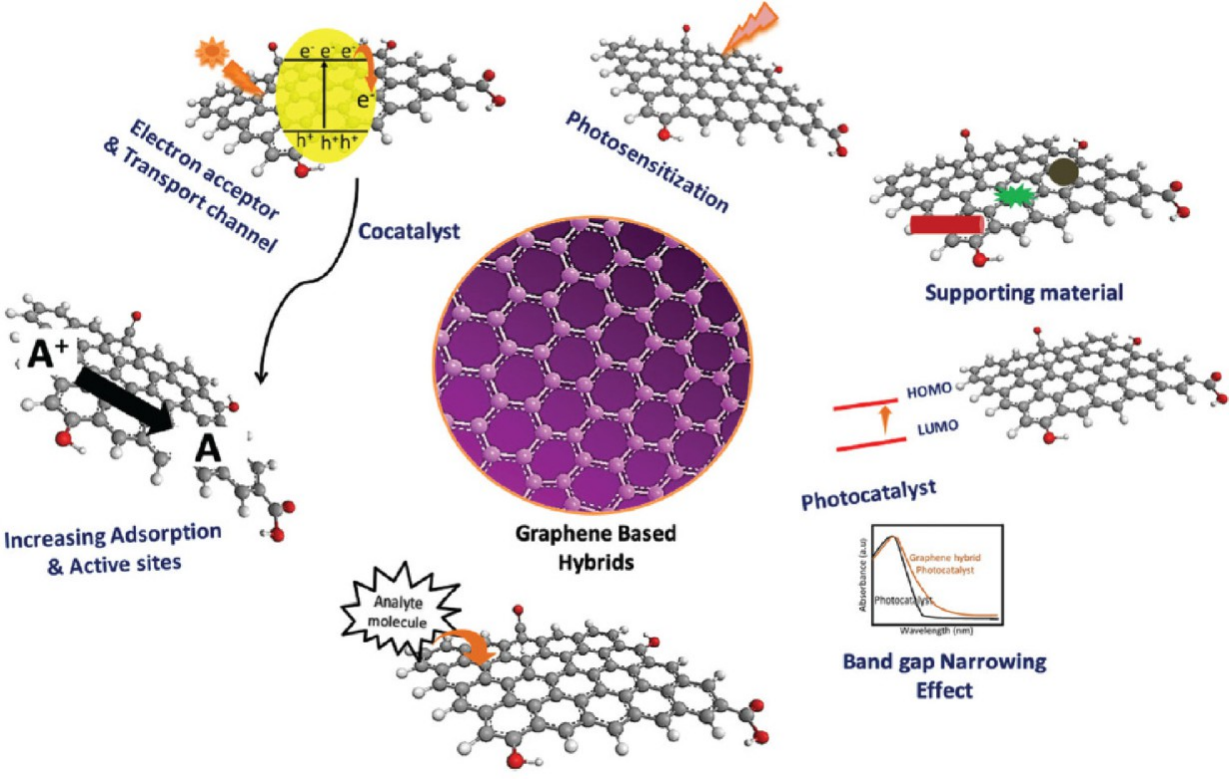
Various roles of graphene in hybrid systems.^136^ Reproduced with permission from ref (136). Copyright 2022 The Royal Society of Chemistry.

Recently, Bhattacharyya et al. presented a detailed explanation on 2D carbon-based combinations that exhibited some exclusive chemical, mechanical, and physical properties that enhanced the productivity of photocatalysis, exclusively with capable photosensitizer combinations.^136^ The main characteristics of the carbonaceous materials are (i) they serve as a template for the deposition of the semiconductor material, (ii) they have a large surface area with excellent stability, (iii) the π-conjugated system facilitates electron transfer, (iv) they enhance the absorptivity through the π–π interaction, (v) the band gap of the semiconductor material can be matched with the 2D carbon materials, (vi) they avoid the aggregation of the semiconductor material, (vii) they improve the recyclability, and (viii) they hinder the electron–hole recombination process. All these properties are shown graphically in Figure 6.

Carbonaceous photocatalysts have increasingly become the standard for PCCR reactions due to their excellent physicochemical and electrochemical properties. Numerous excellent carbon-containing supports, e.g., graphene, CNTs, MWCNTs, CD, and ACFs, etc., have been used for various applications for many years. Carbonaceous compounds have been considered as supports and can be used in many applications, including PCCR for methanol production.

### 2.2 Graphene based catalysts

**Figure 7:**
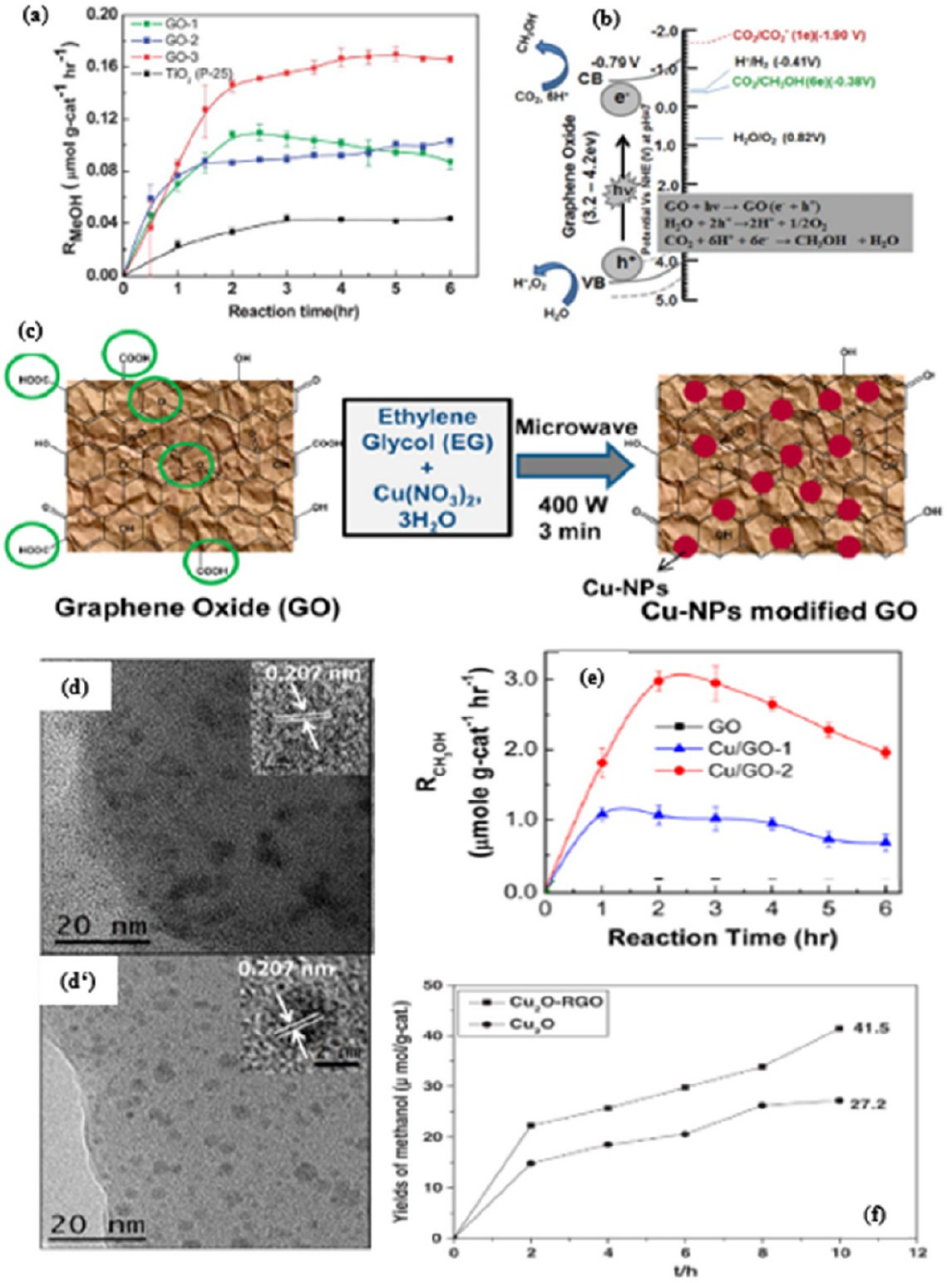
(a) Photocatalytic performance of (GO-1, GO-2, GO-3) and TiO_2_ and (b) schematic illustration of the PCCR mechanism on graphene oxide.^138^ Reproduced with permission from ref (138). Copyright 2013 The Royal Society of Chemistry. (c) Scheme presentation of the microwave synthesis process for GO hybrids decorated with Cu-NPs. (d and d’) Transmission electron microscopy (TEM) images of Cu/GO-1 and Cu/GO-2. Inset: High-resolution TEM (HRTEM) images of single Cu-NP of the respective Cu/GO hybrids. (e) Production rate of methanol on the pristine GO, Cu/GO-1, and Cu/GO-2 as a function of irradiation time,^139^ Reproduced with permission from ref (139). Copyright 2014 American Chemical Society. (f) Methanol yields with the increases of irradiation time, catalyzed by Cu_2_O–RGO nanocomposites and Cu_2_Oparticles.^137^ Reproduced with permission from ref (137). Copyright 2014 Elsevier Inc.

Graphene-based catalysts have recently received increasing attention due to their additional advantages and various applications, including PCCR to CH_3_OH.^79,119^ In 2014, several research groups reported graphene as a photocatalyst for methanol production by CO_2_ reduction.^137−139^ Hsu et al.^138^ synthesized GO by the Hummer method with various GO-1, GO-2, and GO-3 using H_2_SO_4_ and H_3_PO_4_. The synthesized catalysts are used as visible light accessible photocatalysts for PCCR to CH_3_OH, and the schematic representation of PCCR mechanism on graphene oxide is shown in Figure 7a,b. The authors hypothesize that the synthesized modified GO material has an excess of oxygen-containing elements on the basal plane that broaden the band gap energy, which supports the excitation of electrons (e^–^) from VB to CB. It is also proposed that, in PCCR, the photogenerated (e^–^) and (h^+^) migrate to the GO surface and serve as oxidizing and reducing sites, respectively. In another step, the generated (e^–^) and (h^+^) react with the adsorbed CO_2_ and H_2_O on the irradiated GO, resulting in the formation of CH_3_OH, GO generated (e^–^) transfer process, as shown in Figure 7b. It can be concluded that the adapted GO-3 photocatalysts CB and VB have the necessary potential to practically control CO_2_ redox reactions. The reported methanol yield is 0.172 mmol.g_cat_ h^–1^ in visible light, which is 6 times higher than that of pure TiO_2_. In addition, isotopic labeling studies have shown that methanol is formed from CO_2_ and not from photodissociation of GO.

Although materials based on GO are active in PCCR, GO is further modified with copper to increase activity and selectivity. Photocatalytic research on Cu-based catalysts is gaining great interest due to its low cost and large abundance in the Earth’s crust. Shown et al.^139^ reported a simple microwave method for the dispersion of Cu-NP on GO photocatalysts (Figure 7c), which showed a significant improvement in catalytic activity in solar fuel production. The samples prepared by microwave method showed homogeneous dispersion of copper nanoparticles on GO (see Figure 7d,d’). Figure 7e shows the photocatalytic performance of two-dimensionally layered patterned GO nanosheets with a combination of ∼4–5 nm Cu nanoparticles. The improvement in the catalytic performance of Cu/GO is attributed to (i) charge transfer from GO to copper, (ii) electron–hole pair recombination destruction, (iii) band gap reduction of GO, and (iv) work function adjustment. The optimized catalyst (Cu/GO-2) loaded with 10% copper showed excellent performance in producing CH_3_OH (6.84 μmol.g_cat_^–1^.h^–1^) using a visible light source. This was 60 times higher than the unique GO and 240 times higher than the commercially available P-25. Finally, this observation showed a robust cooperation between the metal content in the Cu/GO mixture and the methanol production rate.

In addition to the usual GO doping, much attention has been paid to rGO for PCCR. Another important step related to rGO grafted with semiconductor materials is its unique ability to store and transport electrons *via* electron transfer process, which is very important for photocatalytic reactions. In another report, Wang et al.^137^ synthesized Cu_2_O on rGO by an exclusive *in situ* reduction process using ethylene glycol and copper acetate-adsorbed GO sheets as starting materials. The tested activity for methanol formation by photoreduction of CO_2_ showed good catalytic activity (41.5 μmol.g_cat_^-1^) compared to conventional Cu_2_O catalyst (27.2 μmol.g_cat_^-1^) under simulated sunlight irradiation for 10 h (Figure 7f). The increase in photocatalytic activity of Cu_2_O/RGO is mainly due to charge transfer between Cu_2_O and RGO, enhanced reaction sites, and larger specific surface area. These observations are consistent with the results of Shown et al.^139^

**Figure 8:**
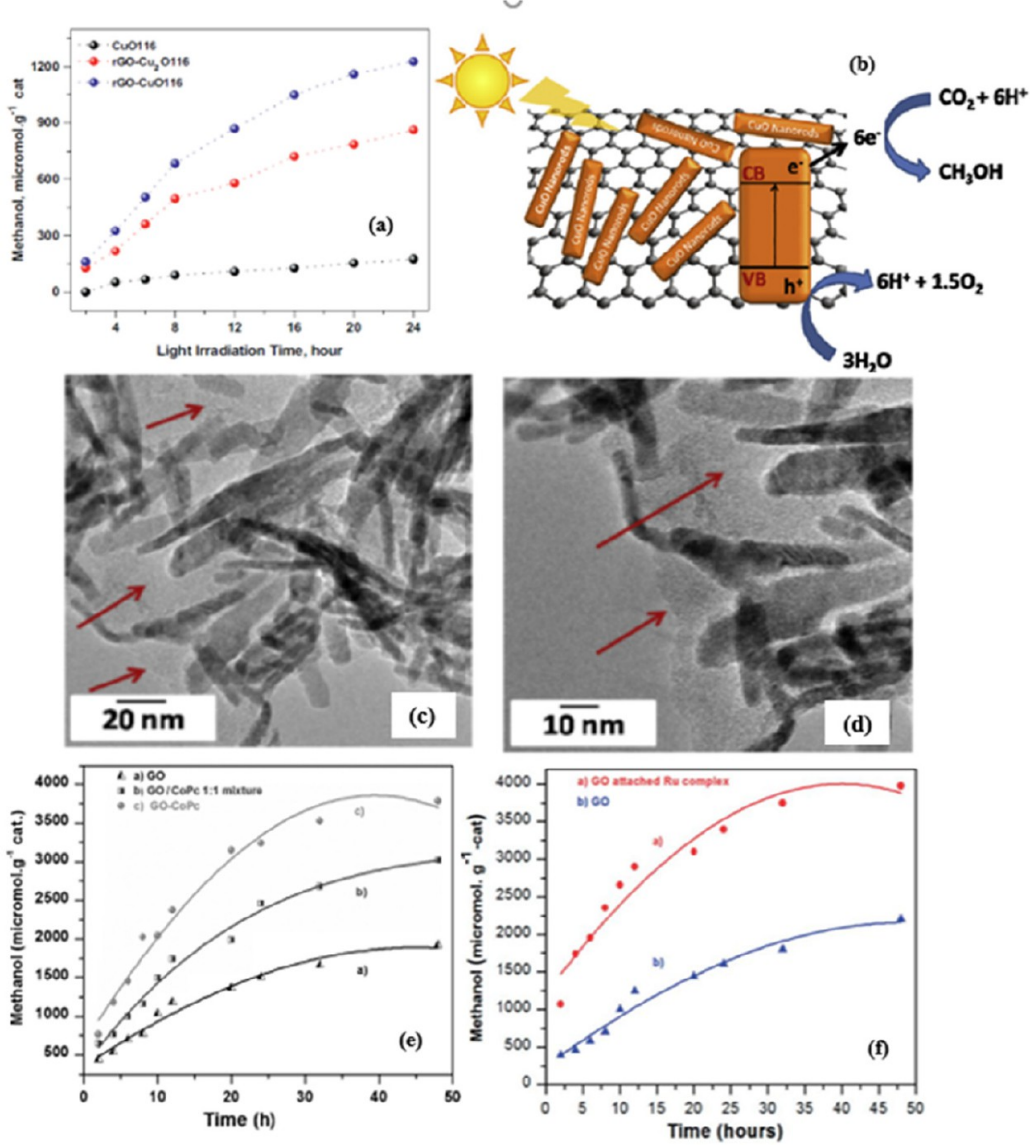
(a) Methanol yield by photocatalytic conversion of CO_2_ as a function of light irradiation time using CuO116, rGO-Cu_2_O116, and rGO-CuO116 nanocomposites, (b) Plausible mechanism of photocatalytic conversion of CO_2_ into the methanol using rGO-CuO nanocomposites under the visible light irradiation. (c and d) HRTEM images of rGO-CuO116 nanocomposites.^140^ Reproduced with permission from ref (140). Copyright 2016 Elsevier Inc. (e) Methanol-conversion rate for (a) GO; (b) GO:Co-Pc (1:1); and (c) GO- CoPc.^141^ Reproduced with permission from ref (141). Copyright 2014 Wiley-VCH Verlag GmbH & Co. KGaA, Weinheim. (f) Conversion of CO_2_ to methanol over time using photocatalysts.^142^ Reproduced with permission from ref (142). Copyright 2014 The Royal Society of Chemistry.

The research group of Kumar and Jain reported three types of catalysts, namely, rGO-CuO,^140^ graphene oxide (GO)-tethered-CoII phthalocyanine combination [CoPc–GO],^141^ and Ru (ruthenium) trinuclear polyazine complex with graphene oxide supported by phenanthroline ligands (GO-phene)^142^ for PCCR to methanol. The copper-based photocatalyst rGO-CuO^140^ was synthesized by covalent fixation of CuO nanorods on rGO material. The photocatalytic activity for methanol yield (Figure 8a) is low (175 μmol.g^–1^) for pure CuO nanorods. In contrast, when rGO-Cu_2_O is used, a drastic increase in activity is observed, i.e., 862 μmol.g^–1^, and in the case of rGO-CuO, the methanol production is 1228 μmol.g^–1^ and these two catalysts provide five- and seven-times higher methanol yields compared to pure CuO, respectively. The high methanol yield of the rGO-CuO catalyst in CuO was attributed to (i) the low recombination of charge carriers, (ii) the coherent movement of the photogenerated electrons through the rGO structure, and (iii) the plausible mechanism of PCCR to CH_3_OH by the nanostructured rGO-CuO material upon visible light irradiation, as shown in Figure 8b. The high-resolution transmission electron microscopy (HRTEM) images of the active catalyst (rGO-CuO116) exhibited a nanorod like structure as shown in Figure 8c,d. Another type of materials is the molecular catalysis of transition metal complexes (CoPc-GO^141^ and Ru-phen-GO^142^), which has also been reported for photocatalytic methanol production from CO_2_ reduction. The main advantages of molecular catalysis are cost efficiency and use as an alternative green photocatalyst, which is favorable for the PCCR system due to the redox reactions with multiple electrons. The author found that the initial homogeneously immobilized metal complexes on the support GO led to electron transfer to CO_2_ in the presence of the metal complex. The yields of methanol for the photocatalysts CoPc-GO and GO-phen were 3781.88 and 3977.57 μmol.g^–1^_cat_ after 48 h of illumination, respectively (Figure 8c,d). The photocatalytic activities of these catalysts are about 1.8 times higher than those of the corresponding untreated GOmaterial. Moreover, the catalysts are reported to be active upon reuse.

**Figure 9:**
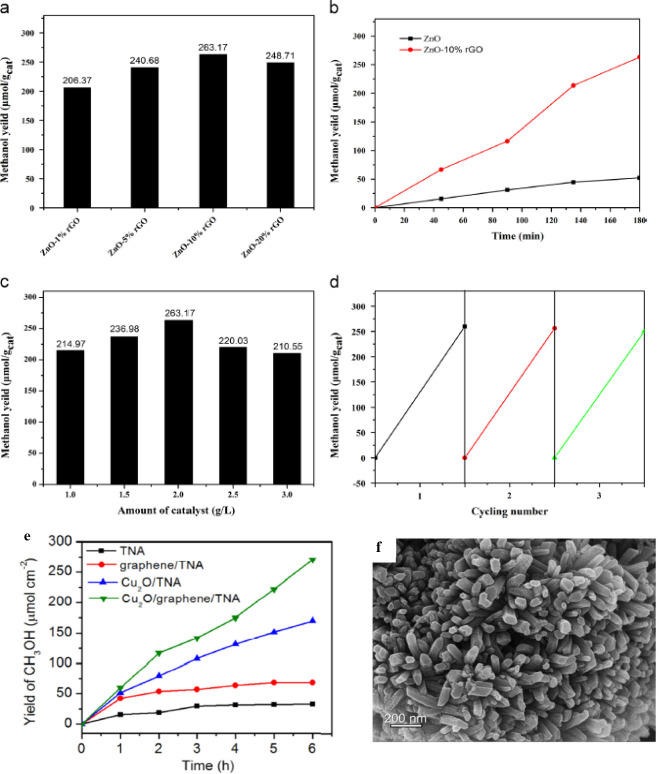
(a) Methanol yield in the photocatalytic reduction of CO_2_ over ZnO-rGO composites with different amounts of rGO under UV–vis light, (b) methanol generation over ZnO-10%rGO and pure ZnO under UV–vis light, (c) effect of amount of photo catalyst on the yield of methanol, and (d) repeated photocatalytic reduction experiments under UV–vis light.^143^ Reproduced with permission from ref (143). Copyright 2015 Elsevier Inc. (e) Photocatalytic methanol evolution over 6 h time course for TNA, graphene-TNA, Cu_2_O/TNA, and Cu_2_O/graphene/TNA catalysts under visible light (λ > 400 nm).^146^ Reproduced with permission from ref (146). Copyright 2016 Elsevier Inc. (f) Field emission scanning electron microscopy (FESEM) images of CNNA/rGO.^144^ Reproduced with permission from ref (144). Copyright 2019 Elsevier Inc.

Zhang et al.^143^ reported a one-pot hydrothermal synthesis process for nanosized zinc oxide on a nanostructured composite of rGO for PCCR to methanol. The ZnO-rGO catalyst showed a methanol yield up to 263.17 μmol.g_cat_^-1^ which is five times higher compared to pure ZnO (Figure 9a,b). It can be seen that the photocatalytic performance of the ZnO/rGO nanocatalyst depends on the amount of catalyst and shows excellent results in cycle tests, as shown in Figure 9c,d. These ZnO-rGO photocatalysts show very good reduction efficiency compared to pure zinc oxide, which is due to the presence of “graphene sheets” in the UV–vis radiation. The authors claim that graphene has a positive effect on the photocatalytic performance of ZnO, which is due to the synergistic effects between ZnO nanoparticles and graphene nanosheets. This synergistic effect can improve the charge separation, which in turn enhances the photocatalytic activity.

A new type of two-dimensional graphene sheets with one-dimensional crystalline CNNA photocatalysts was developed by Xia et al.^144^ by adapting the ionothermal method. The synthesized CNNA/rGO exhibits an ordered composite structure with nanorods arranged perpendicular to the graphene nucleation planes (Figure 9f). The fabricated CNNA/rGO hybrid material exhibits high photoactivity, which can be attributed to enhanced light utilization, efficient exciton splitting, and optimized electron transport pathways. The PCCR product yields are in the following order: CO > methane > ethanol > methanol. The calculated overall quantum yield is 0.254% at 420 nm radiation. Reusability tests with CNNA/rGO were also performed for up to six cycles, and the key observation was that these metal-free catalyst activities were repeatable. These scanning electron microscopy (SEM) images show that CNNA/rGO has an ordered composite structure with nanorod arrangement perpendicular to the graphene nucleation planes (Figure 9f). Moreover, the ordered mesoporous structure of CNNA/rGO improves the CO_2_ adsorption capacity compared to its solid collective form. Another interesting point is that the as-prepared CNNA combined with rGO additionally improves the *Q*_st_ (isosteric heat) value of CO_2_ adsorption to 55.2 kJ mol^–1^. This value exceeds MOFs and porous carbon resources^145^ and allows for high selectivity for CO_2_ photoreduction (87%). The main observation in this case is that this catalyst is composed of nonmetallic active sites and is reusable up to six cycles.

Further improvement in the photocatalytic performance of the ternary composite was observed when it was combined with graphene. This is due to the increased capacity of the photogenerated charge carriers and showed greater catalytic activity for CO_2_ reduction. To further improve the activity in PCCR, the research focus has shifted to ternary compounds. For example, the binary compound Cu_2_O/graphene was modified with titania (Li et al.).^146^ The ternary Cu_2_O/graphene/TNA composite yielded a methanol production rate of up to 45 μmol.cm^–2^.h^–1^ under visible light (Figure 9e). From the comparative study, the order of methanol production rate is as follows: Cu_2_O/graphene/TNA > Cu_2_O/TNA > graphene/TNA > TNA. These results are consistent with the experimental photocurrent density and follow the same trend. The experiment with ^13^C-labeled CO_2_ also confirmed the formation of CH_3_OH from PCCR instead of photoconversion of carbon materials or other origin of carbon in the reduction experiment. They also conducted studies on the strength and activity of the photocatalyst together with pristine graphene. In the study of reusability up to 10 cycles, no significant loss of activity of the Cu_2_O/graphene/TNA catalyst was observed. The authors postulate that the “ternary” arrangement of the photocatalyst shows very good performance and affects the efficiency of the reaction system.

**Figure 10:**
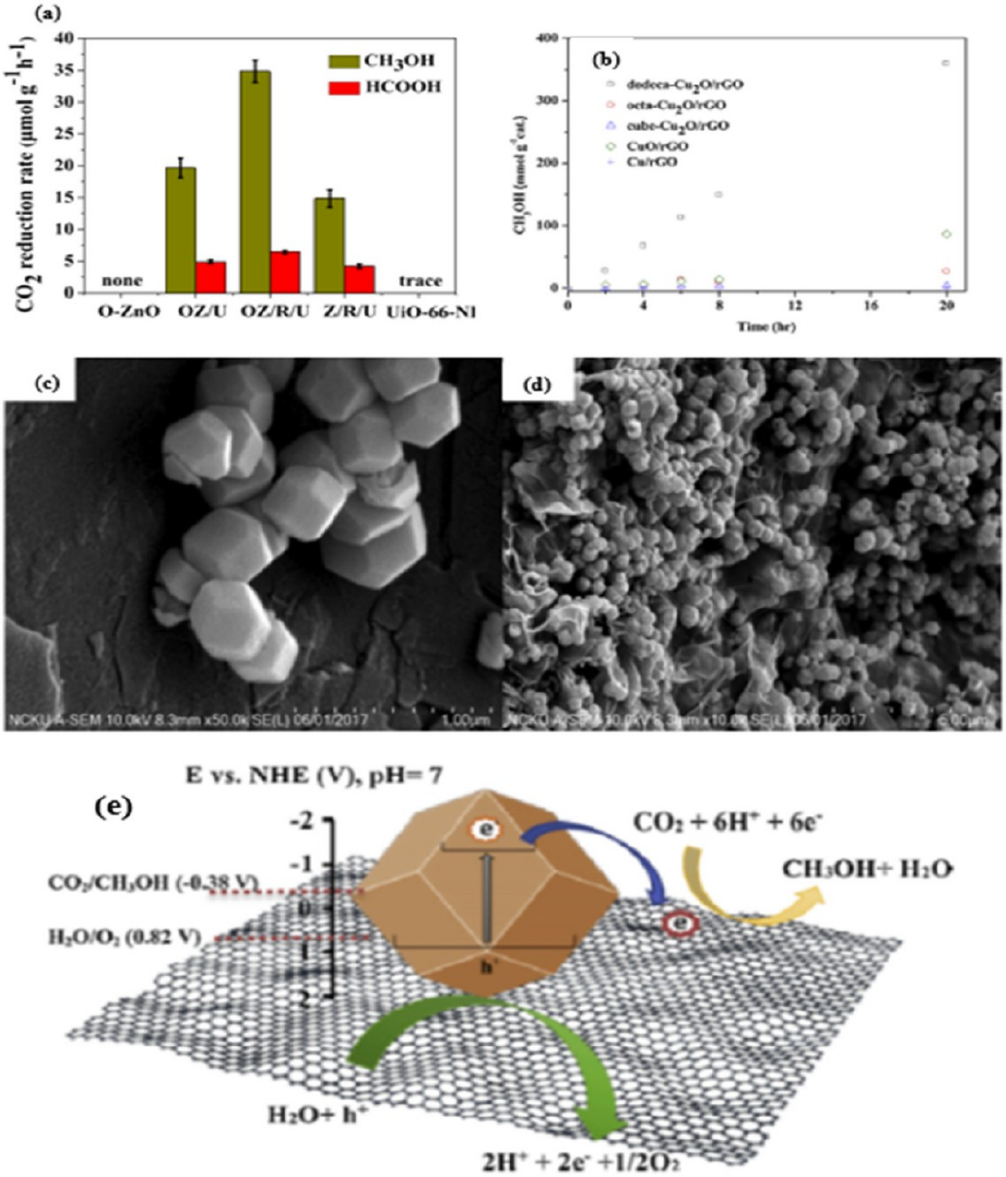
(a) CO_2_ reduction rate over different samples.^147^ Reproduced with permission from ref (147). Copyright 2019 American Chemical Society. (b) Time course of methanol generated by visible irradiation of various photocatalysts. SEM images of (c) dodecahedral Cu_2_O nanocrystals and (d) dodeca-Cu_2_O/rGO and (e) proposed mechanism.^148^ Reproduced with permission from ref (148). Copyright 2019 Elsevier Inc.

Meng et al.^147^ reported that a unique oxygen-defective heterojunction ternary zinc oxide, (O-ZnO)/rGO/UiO-66-NH_2_ (OZ/R/U composite) Z-scheme was synthesized *via* a superficial solvothermal route. The methanol yield (Figure 10a) and formic acid values were 34.83 and 6.41 μmol.g^–1^.h^–1^, respectively, for methanol formation from PCCR (in the presence of visible light). The activity of the synthesized photocatalyst can be explained by the fact that the synthesized OZ/R/U photocatalyst has a high specific surface area, i.e., 877.3 m^2^ g^–1^, so that the ability to adsorb CO_2_ could be enhanced by a large number of active sites for selective CO_2_ photoreduction. Moreover, the incorporation of rGO into the catalyst creates an additional electron bridge, which enables better transfer and separation of photoinduced charges, so that an extreme enhancement of photocatalytic activity is observed. Moreover, the “ternary OZ/R/U” composite exhibited the highest typical fluorescence lifetime, which was attributed to the shift of electrons from the CB = O-ZnO to the VB = UiO-66-NH_2_ by the rGO facilitator. Overall, the Z-scheme system improved the photocatalytic activity due to the suppressed recombination, O-ZnO oxidation ability, and high reduction ability of UiO-66NH_2_.

The next stage of graphene-based photocatalysts are the three-dimensional nanomaterials such as dodecahedron, octahedron, cubic shaped Cu_2_O/rGO nanocatalysts, etc., which have been developed and established for the photoconversion of CO_2_ to CH_3_OH.^148^ The main advantage of the 3D materials compared to the corresponding low-dimensional materials in terms of shape or morphology is the systematized microstructure or even the creation of a 3D network (Figure 10c,d). For example, the Cu_2_O/rGO catalyst with rhombic dodecahedra showed an excellent methanol yield of 355.3 mmol.g^–1^ (Figure 10b) compared to the cubic Cu_2_O/rGO (4.4 mmol.g^–1^), which is due to the lowest band energy gap in the cubic structure. The authors claim that the main reason is the cubic structure of Cu_2_O, which consists of six (100) facets that are photocatalytically inactive sites. Finally, the incorporation of rGO mainly resulted in better transport of photogenerated electrons from CB of Cu_2_O. The authors observed that the photoexited electrons can easily reach the surface of Cu_2_O nanocrystals without recombination of electron–hole pairs. These carbon materials benefit from the generated electrons transferred to the rGO surface and undergo photoreaction with CO_2_ by forming π-electron bonds. In addition, the dodeca-Cu_2_O/rGO photocatalysts are durable and recyclable. Basically, reduced graphene oxide helps as an electron-withdrawing framework. This property holds e^–^–h^+^ pairs to avoid reconnection. A plausible mechanism of CO_2_ photoreduction by dodeca-Cu_2_O/rGO composites is shown in Figure 10e.

**Figure 11:**
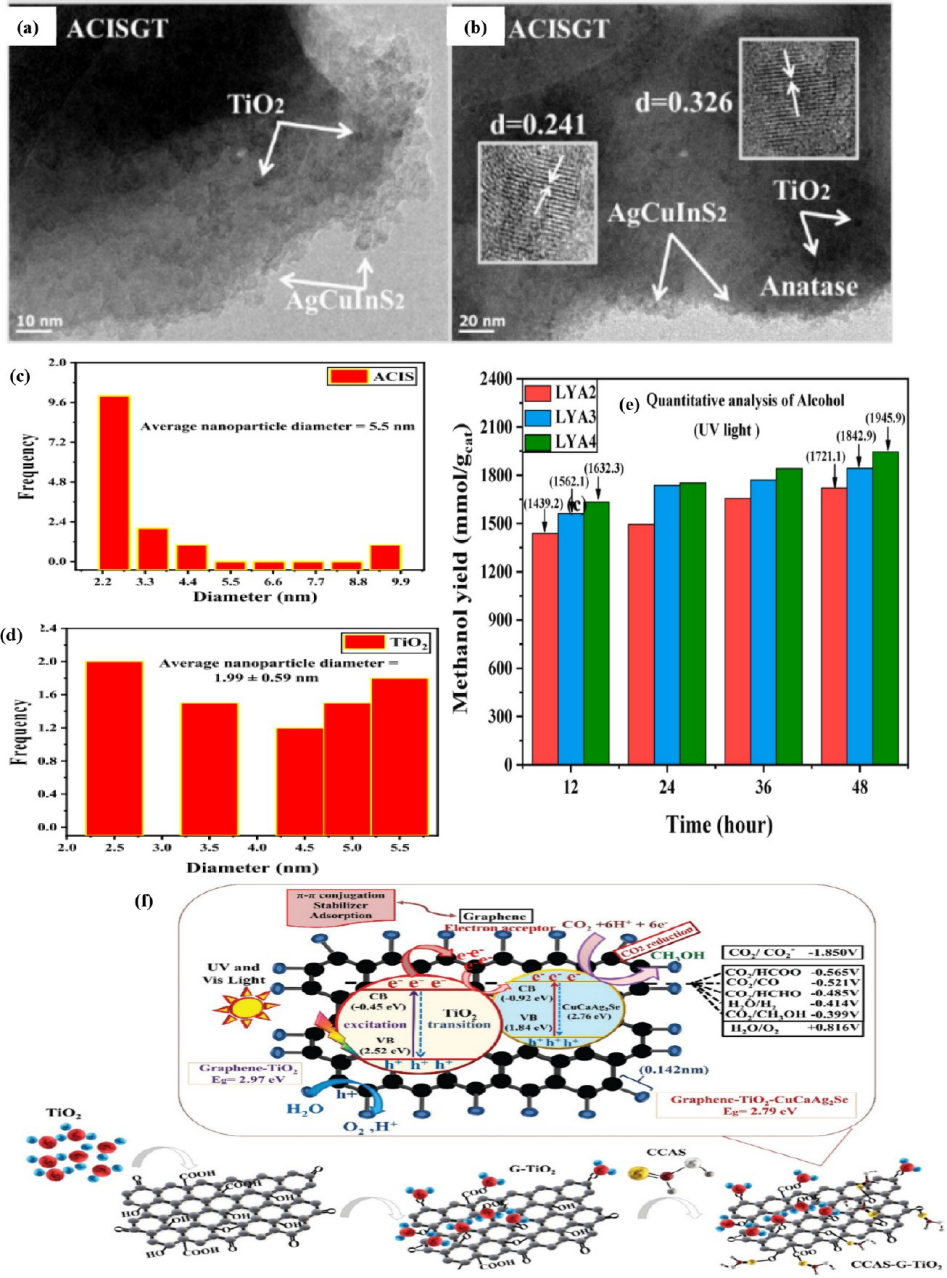
TEM images of (a and b) ACISGT ternary composites and (c and d) nanoparticle size histograms of ACIS and TiO_2_ in ACISG and ACISGT.^149^ Reproduced with permission from ref (149). Copyright 2020 American Chemical Society. (e) Quantitative analyses of alcohol for LYA2, LYA3, and LYA4 nanocomposites under UV light irradiation activity.^151^ Reproduced with permission from ref (151). Copyright 2021 Elsevier Inc. (f) Mechanism and chemical reaction CO_2_ reduction.^150^ Reproduced with permission from ref (150). Copyright 2020 The Royal Society of Chemistry.

In recent years, the research group of Oh and Otgonbayar et al. developed three different graphene-based catalysts (AgCuInS_2_-G-TiO_2_, CuCaAg_2_Se-graphene-TiO_2_ and LaYAgO_4_-graphene-TiO_2_) for the production of methanol by PCCR. Initially, the hydrothermally synthesized ternary nanocomposite material, AgCuInS_2_-G-TiO_2_,^149^ showed a methanol yield of 15.21% using an intercepting agent (48 h). The surface area of graphene was unevenly distributed over the AgCuInS_2_ and TiO_2_ crystals. The standard sizes of AgCuInS_2_ and titania as prepared are about 5.5 and 1.99 nm, respectively (Figure 11a–d). Compared to the binary (AgCuInS_2_-G) and ternary (AgCuInS_2_-G-TiO_2_) compounds, the ternary catalyst exhibited exceptional stability and photocatalytic activity up to four cycles. It is reported that AgCuInS_2_ and graphene are activated in parallel upon light irradiation, and the e^–^ transfer from the graphene moiety reaches the VB of titania to form the donor level.

Second, the CuCaAg_2_Se-graphene-TiO_2_ (CCAS-G-TiO_2_) ternary composite^150^ was synthesized by a hydrothermal muffle accommodation method. The yield of ethanol in the photoreduction of CO_2_ is 12.68 (under visible light) and 16.84% in the presence of UV light (48 h, scavenger 0.6 g). This is about 6-fold higher photoactivity compared to titania, pure CuCaAg_2_Se, and the binary nanocatalyst. The authors note that graphene acts as a link between the CCAS and titania, improving charge separation and also increasing the capacity of the photogenerated charge carriers. This ternary CCAS-G-TiO_2_ compound is also recyclable for up to nine intervals (432 h) under UV–vis light irradiation and 0.3 g of scavenger. The main role of TiO_2_ is that it is bonded to the graphene materials with a “sheet-like” arrangement and can act as an electron integrator in facilitating the PCCR reaction mechanism (Figure 11f). The authors describe that graphene plays a central role as an electron acceptor/transporter and is the most important part of the (e^–^) and (h^+^) transport distribution.

Finally, another graphene-based ternary nanocomposite, LaYAgO_4_-graphene-TiO_2_,^151^ was prepared and tested for the PCCR reaction. The yield of methanol is 1945.9 mmol.g_cat_^–1^ (under UV light) after 48 h (Figure 11e). In the presence of UV–vis light, graphene and LaYAgO_4_ are excited equivalently, and electrons are transfers from the VB of titania through the graphene to produce a donor level. In conclusion, graphene plays a central role in the electron transfer mechanism in all synthesized catalysts. Moreover, the authors found that all the above heterojunction catalysts provide a very capable route for the development of breakthrough photocatalytic materials for various applications in different sectors, such as environmental protection and solar energy conversion.

**Figure 12:**
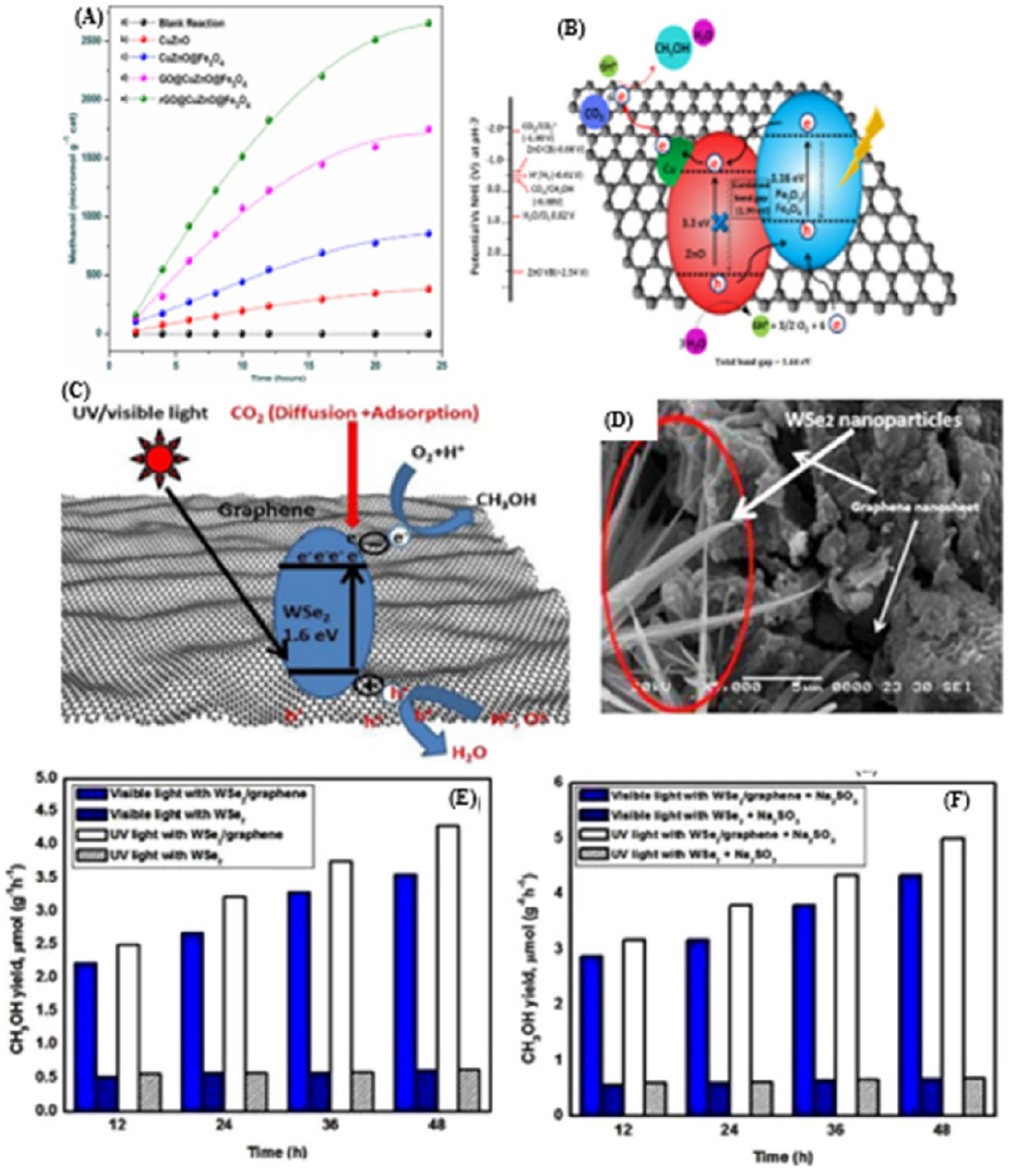
(A) Yield of methanol over Cu, Zn-based catalysts: (a) blank reaction, (b) using 1 wt % CuZnO, (c) CuZnO@Fe_3_O_4_, (d) GO@CuZnO@Fe_3_O_4_, and (e) rGO@CuZnO@Fe_3_O_4_. (B) Plausible mechanism of CO_2_ reduction by rGO@CuZnO@Fe_3_O_4_.^152^ Reproduced with permission from ref (152). Copyright 2017 Elsevier Inc. (C) Mechanism study of photocatalytic reduction of CO_2_. (D) SEM image of WSe_2_-graphene composite. Methanol yields in the photocatalytic reduction of CO_2_ under UV–visible light irradiation by using pure WSe_2_ and WSe_2_-graphene nanocomposites as photocatalyst (E) without Na_2_SO_3_ and (F) with Na_2_SO_3_.^155^ Reproduced with permission from ref (155). Copyright 2017 Springer Nature.

Kumar et al.^152^ reported a novel magnetically separable rGO@CuZnO@Fe_3_O_4_ microsphere photocatalyst with a core–shell structure that enabled the reduction of CO_2_ upon visible light illumination and provided a methanol yield of 2656 μmol.g^–1^. The plausible mechanism for CO_2_ reduction with rGO@CuZnO@Fe_3_O_4_ is shown in Figure 12A,B. As discussed for other catalytic systems, the absence of rGO in the catalyst (CuZnO@Fe_3_O_4_) showed a very low methanol yield (858 μmol.g^–1^ cat), clearly indicating the importance of rGO for PCCR for selective methanol formation. This is due to the special properties of rGO, such as its higher electron association and charge separation over the rGO aromatic system. Reusable and highly capable photocatalysts for PCCR to methanol were observed in the presence of rGO, and noted capabilities included a large surface area and excellent charge carrier mobility. The copper content also influenced the methanol yield, with the optimum Cu content being 1%. The main role of Cu content in methanol production is to trap photogenerated electrons, leading to a reduction in e^–^–h^+^ pair recombination. Another major advantage of the rGO@CuZnO@Fe_3_O_4_ catalyst is its magnetic separability after completion of the photoreaction. The reusability of the catalyst up to six series showed stable activity, and the yield of methanol was almost identical for all cycles. Therefore, the as-synthesized rGO@CuZnO@Fe_3_O_4_ catalyst is cheap, has superior electron mobility, and is more environmentally friendly than ZnO.

In addition, there are few reports on various metal/metal oxide-based photocatalysts, which are discussed in the following section. Nanomaterials of titania-graphene (TiO_2_-rGO) prepared by a simple chemical technique are also used for the photoreduction of CO_2_ to CH_3_OH with a formation rate of 2.2 μmol.g^–1^.h^–1^.^153^ The main conclusion of the authors is that the efficiency of photocatalytic performance increases with an increase in graphene content up to 50%, with methane and methanol being the main products. It is postulated that graphene materials increase the separation efficiency of photogenerated electrons, possess the most active adsorption sites, and thus increase the number of photocatalytic reaction centers. Silver and tungsten selenide-based catalysts (Ag_2_Se-G-TiO_2_, WSe_2_ graphene) were also used for the PCCR to methanol. The nanoscale Ag_2_Se-G-TiO_2_ photocatalyst^154^ gave a total methanol yield of 3.5262 μmol.g^–1^.h^–1^ after 48 h in the presence of UV–visible light. The individual components of pristine TiO_2_ and Ag_2_Se-graphene nanosheets showed a very low photoreduction rate compared to Ag_2_Se-G-TiO_2_ nanocomposites. This is because graphene is the main component of the catalyst, as discussed previously for other catalytic systems, i.e., graphene acts as an interfacial contact between Ag_2_Se and TiO_2_, enhancing the synergistic effect between the two and affecting the overall photoreduction activity.

The photocatalytic system with nanowire structure of WSe_2_-graphene^155^ showed a methanol yield of 5.0278 μmol.g^–1^.h^–1^ in the presence of UV and visible light. The activity of WSe_2_-graphene can be attributed to its properties. The reusability of the WSe_2_-graphene was investigated for six consecutive cycles, and there was no significant loss of methanol yield, so the photocatalyst was more stable and could be useful for a continuous PCCR system. It is also clear from the above sections that the graphene features such as (i) a large surface area, (ii) multiple defective positions for CO_2_ absorption, and (iii) photogenerated electrons on the WSe_2_ are transferred to the graphene active centers, and at this point, the reduction of absorbed CO_2_ is converted to methanol. The authors proposed a photocatalytic mechanism, and SEM images of the WSe_2_-graphene composite are shown in Figure 12C,D. As shown in Figure 12C, when the WSe_2_ nanomaterial is irradiated with light from the solar spectrum, photogenerated charge carriers (holes and electrons) are generated. The graphene in the catalyst separates the electron–hole pairs and acts as a transporter in the binary system. Thus, the photoinduced holes on the WSe_2_-g VB absorb water molecules and form hydroxyl radicals (OH^•^), which then further oxidize the protons (H^+^) and oxygen. Meanwhile, electrons are transferred and absorbed by CB CO_2_ to form ^•^CO^–^_2_. The ^•^CO^–^_2_ reacts with the ^•^H radical, which then leads to the formation of a series of radicals and finally forms CH_3_OH.

Another Ta_2_O_5_-based photocatalyst modified with rG was developed by Lv et al. using a one-step hydrothermal technique.^156^ The catalyst with 1% graphene exhibited a maximum methanol production that was 3.4 times higher than that of the catalyst without graphene. This shows the importance of graphene in supporting the collection and mobility of multiple electrons in the reduction of carbon dioxide. It was also found that the addition of NiO promoter to the Ta_2_O_5_-rGO material further enhanced the photocatalytic activity (high yield of CH_3_OH and H_2_).

### 2.3 Carbon Nanotube Catalysts

**Figure 13:**
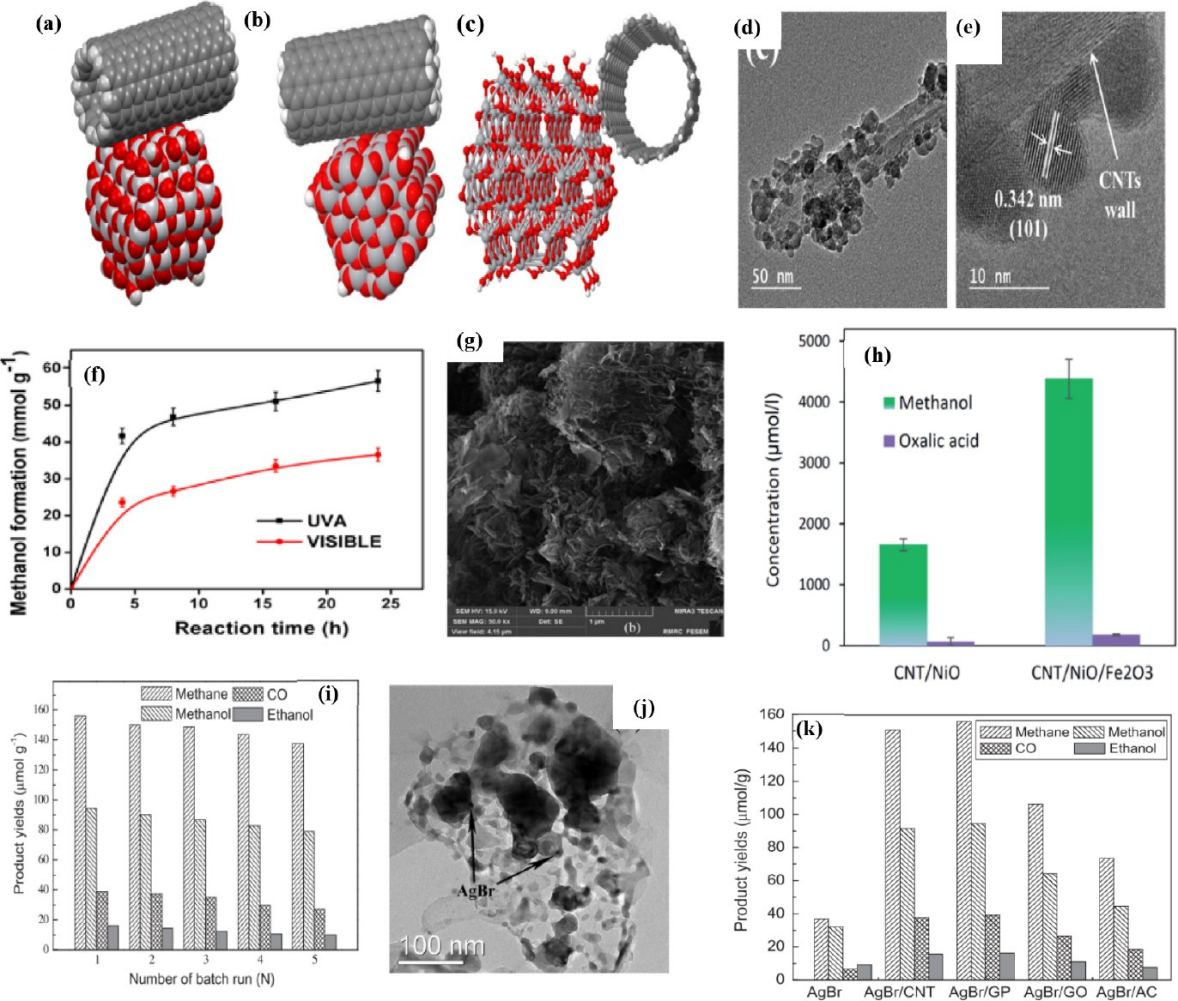
CNT-TiO_2_ composite structure with CNT attachment to the (a) (001) facet and (b and c) (101) facet. (d and e) TEM and HRTEM images of the synthesized 2.0CNT-TiO_2_. (f) Time on stream yields of methanol over 2.0CNT-TiO_2_ under UVA and visible light in the ACN/H_2_O/TEOA medium.^157^ Reproduced with permission from ref (157). Copyright 2019 American Chemical Society. (g) SEM image of CNT/NiO/Fe_2_O_3_ nanocomposite materials under consideration. (h) Power of photocatalysts to convert CO_2_ into methanol and oxalic acid (the main liquid-phase byproduct).^158^ Reproduced with permission from ref (158). Copyright 2020 The Royal Society of Chemistry. (i) Product yields for AgBr/GP nanocomposite number of batch runs. (j) TEM images of AgBr/GP. (k) Product yields for different carbon-based AgBr nanocomposites.^159^ Reproduced with permission from ref (159). Copyright 2014 Elsevier Inc.

Similar to GO and rGO, CNTs enable enhanced photocatalytic performance of immobilized catalysts. Kumar et al.^157^ reported a series of CNT-TiO_2_ nanocomposites for PCCR and H_2_O splitting reactions under both UV-A and visible light. The authors investigated the effects of CNTs on the morphology of TiO_2_, and HRTEM shows that the anatase TiO_2_ plane (101) has a lattice spacing of 0.342 nm. X-ray diffraction (XRD) and surface analysis investigated the influence of CNTs on TiO_2_, and the results show that the increase in CNT content decreases the crystallinity of the CNT-TiO_2_ nanocomposite. From computational studies, when CNTs are bonded to titania NPs, the desired plane is (101) instead of (001) planes. The authors reported that, in the case of visible light, charge transfer from CNTs to TiO_2_ occurs through the buildup of isolated charge carriers. In contrast, in the case of UV light, the charge transfer occurs in two directions, from TiO_2_ to CNTs and from CNTs to TiO_2_ (Figure 13a–e). The active nanocomposition 2.0CNT-TiO_2_ was able to achieve the highest yields of CH_3_OH, H_2_, and formic acid in PCCR. The corresponding yields were 2360.0, 3246.1, and 68.5 μmol.g^–1^.h^–1^ (Figure 13f). The authors also demonstrated that the photocatalytic stability increased with the addition of CNTs in the CNT-TiO_2_ nanocomposite.

One of the applications of CNTs is hydrogen storage capacity, which is used for the conversion of CO_2_ by photoreduction^158^ using CNT/NiO and CNT/NiO/Fe_2_O_3_ composites synthesized by a simple hydrothermal method over earth-rich elements for PCCR to methanol. The author also found that CNTs play an important role in photocatalytic applications (CO_2_ to alcohols) and that CNTs can also store hydrogen. The morphology study for the prepared catalyst shows a porous nanoflake or flower-like structure in which carbon nanotube fibers were dispersed in the nanocomposite material using an identical process (Figure 13g). Both the binary and ternary composites are active in the preparation of methanol. However, the addition of Fe_2_O_3_ to the binary composite resulted in 2.6 times higher methanol production than the CNT/NiO binary composite (Figure 13h). The addition of Fe_2_O_3_ to CNT/NiO decreased the system impedance, and the surface area and ability to effectively utilize incident photons were also increased.

Fang et al.^159^ reported that AgBr nanocompounds were deposited on various carbon-based support materials, i.e., MWCNT, GP, EG, AC, etc., by the deposition-precipitation technique using CTAB. PCCR efficiency was investigated in the presence of visible light. Using XRD and TEM, the authors demonstrated that the AgBr nanoparticles were well-dispersed on the support materials (Figure 13j). It is reported that the AgBr/CNT and AgBr/GP catalysts exhibited a moderately higher yield of methanol (94.44 μmol.g^–1^) under visible light, which was attributed to the transfer of photoexcited e^–^(electrons) from the CB of AgBr to carbon as the support material (Figure 13k). The active catalytic efficiency AgBr/GP decreased to 83% after five repeated runs, while the overall photocatalytic yield was 83% in the first run. In the case of the AgBr catalyst, the yield decreased by 6% after three cycles of use compared to the first run (Figure 13i).

**Figure 14:**
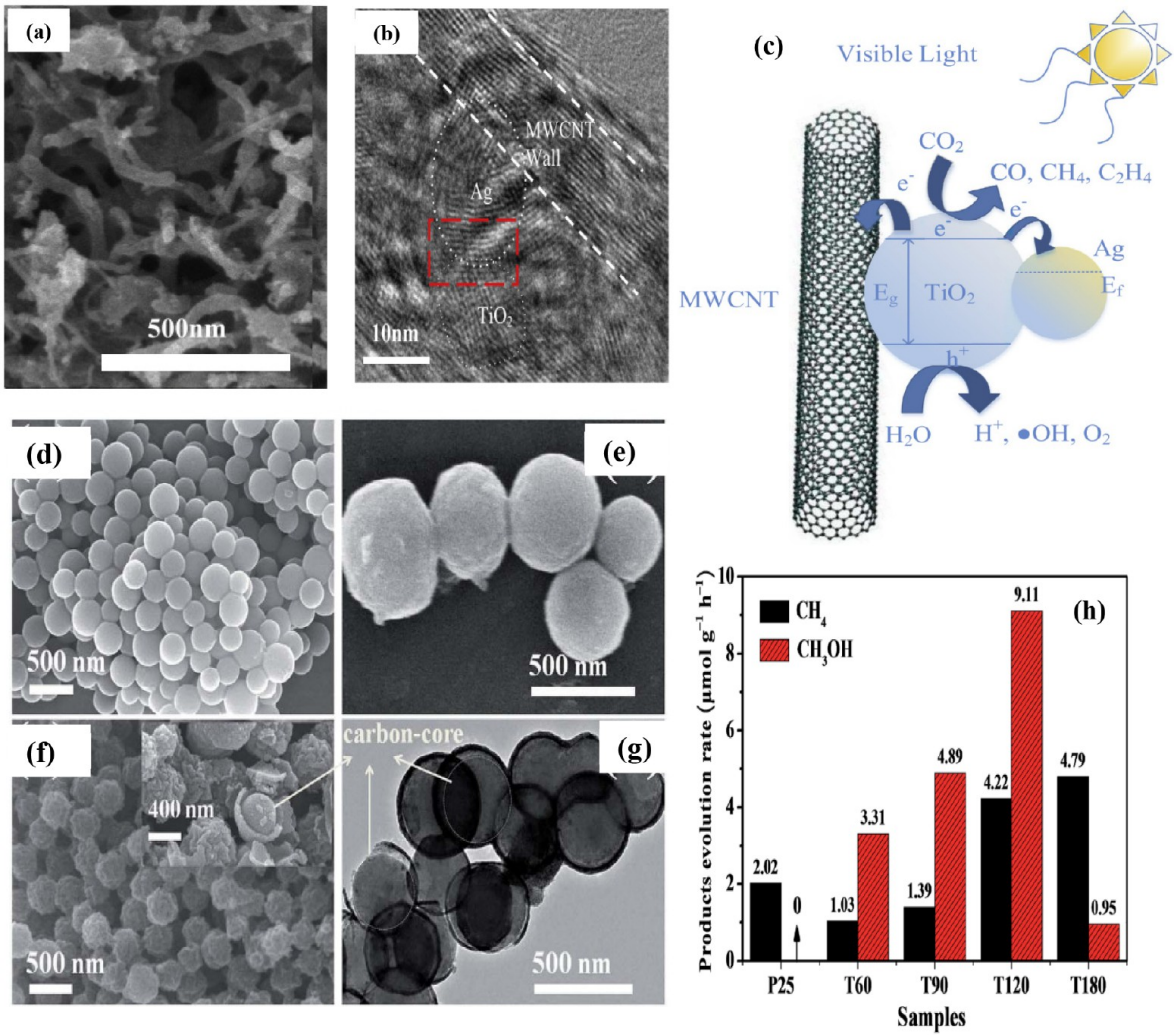
(a) FESEM and (b) HRTEM images of 2 wt % Ag-MWCNT@TiO_2_. (c) Proposed schematic of charge transfer and reactants transformation using Ag-MWCNT@TiO_2_.^160^ Reproduced with permission from ref (160). Copyright 2015 Elsevier Inc. FESEM images of typical samples (d) CNS, (e) CSTS, and (f) T60. (g) TEM images of T60. (h) Comparison of the photocatalytic CH_4_ or CH_3_OH evolution rate of carbon@TiO_2_ composite samples and P25 (under simulated solar light).^162^ Reproduced with permission from ref (162). Copyright 2017 The Royal Society of Chemistry.

Gui et al.^160^ successfully synthesized Ag-MWCNT@TiO_2_ nanocomposites with a core–shell structure that exhibited excellent visible light accessible properties for PCCR applications. Low Ag loading (2%) does not effect on the surface morphology (Figure 14a,b), as it looks similar and identical with that of MWCNT@TiO_2_. This is due to the incorporation of the Ag nanoparticles into the close-packed arrangement of the nanotitania and not due to the random scattering on the surface of the nanotube. The catalytic performance for CO_2_ photoreduction showed 6.34 μmol.g_cat_^-1^and 0.68 μmol.g_cat_^-1^ toward CH_4_ and C_2_H_4_, respectively. Figure 14c shows the charge transfer mechanism in Ag-MWCNT@TiO_2_.

In addition, cobaltphosphide on carbon-based material is used for the PCCR of CO_2_.^161^ The combined CoP/CNT and CoP/rGO hybrid photocatalysts were prepared by a simple, scalable technique in a hydrothermal autoclave reaction. The performance and selectivity of the two synthesized photocatalysts are superior to those of CoP in bulk. The PCCR studies showed that graphene and CNT exhibit high photocatalytic conversion of CO_2_ due to the strong interactions between CoP and carbon supports. The main interaction between CoP and carbonaceous resources (CNT/rGO) is affected by P defects on the CoP surface and partially adsorbed H_2_O fragments, which reduce the activation energy of RDS. CO catalytic rate and selectivity were absolutely indicated by experimental results and calculation statistics.

Carbon@TiO_2_ hollow spheres (Figure 14d–g)^162^ were also prepared from colloidal carbon spheres, and this composite was used for photoreduction of CO_2_. The authors reported that the methanol formation rate was 9.1 μmol.g^-1^.h^-1^ (Figure 14h) over carbon@TiO_2_, which is better than pristine TiO_2_. FESEM, TEM, and STEM analyses showed that the as-synthesized photocatalyst materials had a hollow, spherical structure, and elemental mapping was performed (Figure 14d–g). From the EI spectra, it was found that the carbon content in the as-synthesized carbon@TiO_2_ catalyst affected the charge transfer ability. The main factors affecting the activity of carbon@TiO_2_ in the photoreduction of CO_2_ are the increase in specific surface area (110 m^2^/g) due to the presence of carbon, which increases the absorptivity of carbon dioxide, and the intrinsic photothermal influence near the photocatalyst due to carbon. It was also found that the carbon@TiO_2_ composite photocatalyst has very good CO_2_ reduction performance compared to the TiO_2_-graphene and TiO_2_-multiwalled carbon nanotube nanocomposites.

### 2.4 Activated Carbon Fiber Catalysts

**Figure 15:**
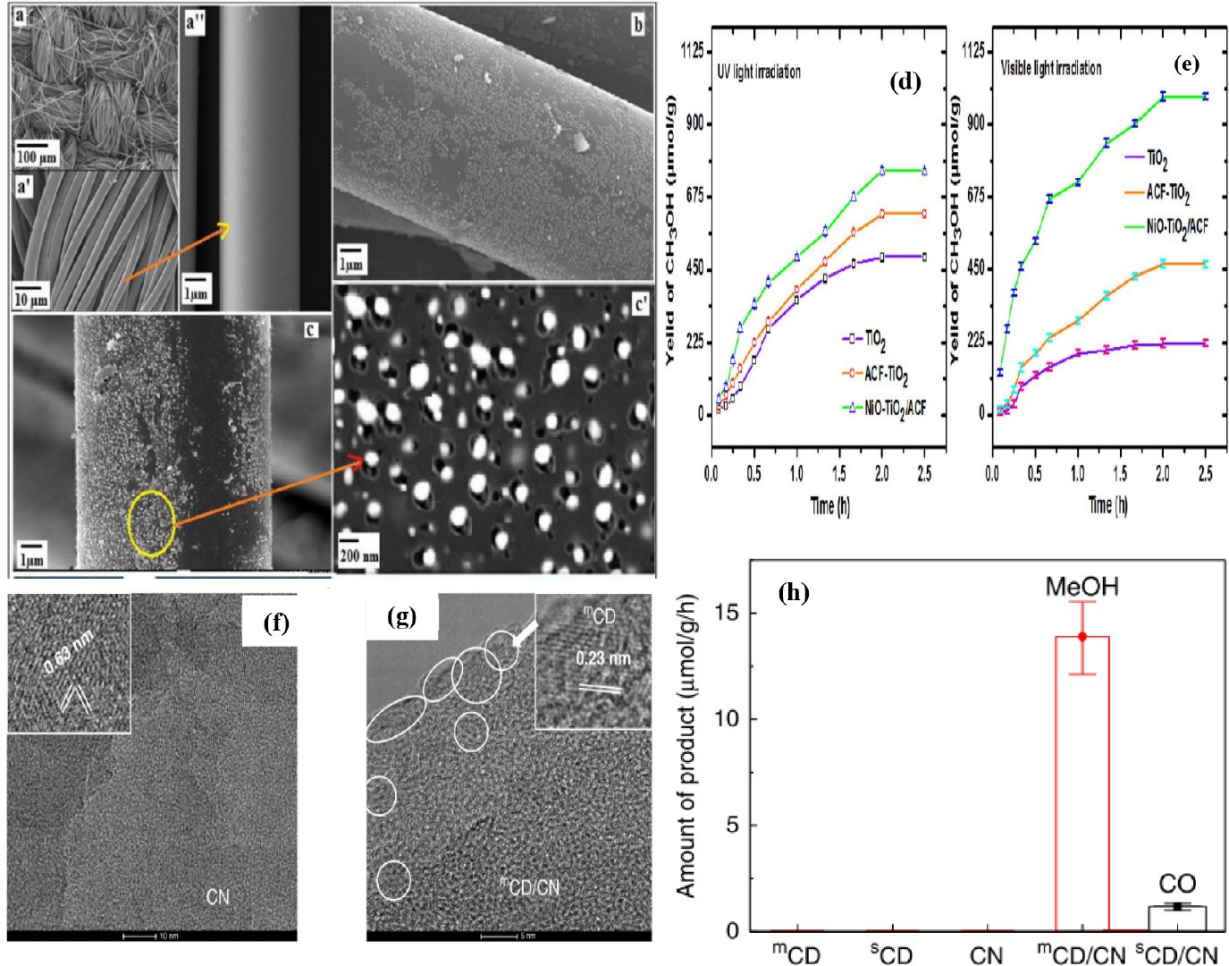
SEM of (a–a”) ACF, (b) ACF-TiO_2_, and (c and c’) NiO-TiO_2_/ACF. Conversion of CO_2_ into methanol under the presence of (d) UV and (e) Visible light irradiation.^163^ Reproduced with permission from ref (163). Copyright 2017 Elsevier Inc. (f) HRTEM images of CN. Scale bar: 10 nm. Inset: an enlarged image showing (110) crystal fringes of CN. (g) HRTEM images of ^m^CD/CN nanocomposite. Scale bar: 5 nm. ^m^CD are marked by circles. Inset: an enlarged image showing graphite superstructure of ^m^CD. (h) Control experiments on ^m^CD, ^s^CD, CN, ^m^CD/CN, and ^s^CD/CN. Error bar: ^m^CD/CN 13.9 ± 1.7 μmol.g^-1^.h^-1^, ^s^CD/CN 1.2 ± 0.2 μmol.g^-1^h^-1^.^164^ Reproduced with permission from ref (164). Copyright 2020 Springer Nature.

Sharma et al.^163^ reported the synthesis of a nanocomposite of NiO-TiO_2_/ACF as a photocatalyst for the production of methanol from CO_2_ by the sol–gel method. The NiO-TiO_2_/ACF composite produced 755.1 and 986.3 μmol.g^-1^ in the presence of UV and visible light, respectively (Figure 15d,e). The mechanism involved in the production of methanol is the transfer of e^–^–h^+^ pairs (photogenerated charge carriers) to the NiO/TiO_2_ surface, which are involved in the reduction/oxidation processes. This indicates the importance of activated carbon fibers for photocatalytic performance, as they exhibit higher activity than Ni loaded titania (e^–^–h^+^ recombination is inhibited by ACF). Moreover, activated carbon fibers (surface area: 163.9 m^2^/g) and NiO increase the ability to adsorb CO_2_ and also modify the electronic absorption properties of TiO_2_. The as-prepared ACF, ACF-TiO_2_, and NiO-TiO_2_/ACF photocatalysts were detected by SEM, XRD, and X-ray photoelectron spectroscopy (XPS) (Figure 15a–c’): The titania NPs were homogeneously deposited on the surface of the ACF material with excessive purity and crystallinity. It can be seen that the NiO-TiO_2_/ACF photocatalyst is still strong after 10 cycles and exhibits exceptional photocatalytic activity for PCCR.

### 2.5 Carbon-Dots/Carbon Nitride Catalysts

Wang et al.^164^ investigated the excellent variability of the combination of carbon-dots/carbon nitride (^m^CD/CN)-based photocatalysts using a flexible microwave technique (^m^CD, graphite phase) and used it to produce CH_3_OH from CO_2_. The surface morphology of ^m^CD and CN was investigated using HRTEM, and CN exhibited hexagonal lattice fringes (Figure 15f,g). The individual components, i.e., ^m^CD and pure CN, did not showed photocatalytic reduction of CO_2_. However, the ^m^CD/CN nanocomposite was active in CO_2_ photoreduction. In contrast, the physical mixture of ^m^CD and carbon nitride showed no significant activity. These active photocatalysts were tested for recyclability in three consecutive runs. No significant changes were observed, indicating the outstanding strength of the nano^m^CD/CN composite. The authors also performed the confirmation test for the carbon source in the PCCR experiment of ^13^C-labeled CO_2_ using the ^m^CD/CN photocatalyst. Moreover, the particular ^m^CD holes were trapped by CN and hindered CH_3_OH adsorption, which affected the oxidation of H_2_O instead of methanol and increased the selectivity of alcohol (Figure 15h). The PCCR to methanol using various carbonaceous materials discussed in the previous sections are listed in Table 3.

**Table 3:** Photocatalytic CO_2_ Reduction for the Various Graphene Based and Carbonaceous Materials

S. No.	photocatalyst	light source	reaction time (h)	catalyst preparation	products CH_3_OH yield	year	ref.
1	GO-3	30 W halogen lamp		modified hummers’method	CH_3_OH (0.172 mmol.g_cat_^–1^.h ^–1^)	2013	(138)
2	Cu-GO-2	300 W halogen lamp		simple microwave process	(2.94 μmol.g_cat_^–1^ h^–1^)	2014	(139)
3	Cu_2_O-RGO	500 W Xe lamp	10	*in situ* reduction method	CH_3_OH 41.5 μmol.g _cat_^-1^	2014	(137)
6	ZnO/RGO	300 W Xe lamp	3	one-pot hydrothermal process	263.17 μmol.g_cat^-1^_	2015	(143)
7	rGO-CuO	20 W white LED	24	covalent grafting method	1282 μmol.g^–1^	2016	(140)
8	Cu_2_O/graphene/TNA	300 W Xe lamp (λ>400 nm)		sequential electrochemical deposition	45 μmol.cm^–2^.h^–1^	2016	(146)
9	CNNA/rGO	350 W Xe lamp, AM1.5		ionothermal method	0.53 μmol.g_cat_^–1^.h^–1^	2019	(144)
10	Cu_2_O/rGO	300 W Xe lamp (λ > 420 nm)	20	solution-chemistry route	355.3 mmol.g^–1^	2019	(148)
11	O-ZnO/rGO/UiO-66-NH_2_	300 W Xe lamp (λ > 420 nm)		solvothermal method	34.83 μmol.g^–1^.h^–1^	2019	(147)
12	AgCuInS_2_-G-TiO_2_	Halide lamp of 500 W	48	hydrothermal method	15.21%^a^	2020	(149)
13	CuCaAg_2_Se-graphene-TiO_2_	Metal halide lamp (500 W)	48	muffle-assisted hydrothermal method	16.84%^a^	2020	(150)
14	LaYAgO_4_-graphene-TiO_2_	Halide lamp of 500 W	48	hydrothermal method	12.27%^a^ (1945.9 mmol.g_cat_^–1^)	2021	(151)
15	rGO@CuZnO@Fe_3_O_4_	20 W white cold LED light (λ > 400 nm)	24	hydrothermal method	2656 μmol.g_cat_^–1^	2017	(152)
16	TiO_2_-RGO	high-pressure mercury lamp (250W)		simple chemical method	2.2 μmol.g^–1^.h^–1^	2016	(153)
17	Ag_2_Se-G-TiO_2_	metal halide lamp (500 W)	50	ultrasonic techniques	3.5262 μmol.g^–1^.h^–1^	2017	(154)
18	WSe_2_-graphene	metal halide lamp (500 W)	48	ultrasonication and	5.0278 μmol.g^–1^.h^–1^	2017	(155)
19	NiO_*x*_-Ta_2_O_5_-rG	500 W Xe lamp	48	hydrothermal method	20 μmol	2013	(156)
20	rGO-NH2-MIL-125(Ti)	20 W visible light white cold LED lamp	24	**-**	47.2 mmol.g^–1^	2020	(165)
21	STO/Cu@Ni/SiO_2_	300 W Xe lamp	4	coprecipitation.	76.9 μmol.g^–1^	2023	(166)

a Represented in percentage.

Overall, the addition of carbonaceous materials to the photocatalyst has been shown to increase product yield. This is due to the synergistic effect between the carbonaceous materials and the catalyst. The addition of carbonaceous material to the photocatalyst results in extensive π–π conjugation, which increases (i) the circulation of photoexcited electrons captured by the photocatalyst, (ii) the CO_2_ adsorption capacity, (iii) the response to visible light, and (iv) the increase in surface area. The combination of carbon with photocatalysts results in a variety of multicomponent heterostructures that enhance charge separation and transport, extend the lifetime of charge carriers, and increase their photocatalytic activity. Since carbon is affordable, environmentally friendly, and sustainable, it has the potential to be used as a photocatalyst on a large scale and in industry.

## 3 Chemical Reaction Kinetics of CO_2_ Photo Reduction

Kinetics studies for each reaction are important to understand the mechanism and help in further steps from batch-scale to large-scale optimization processes. In this section, only the basic concepts are presented and discussed based on the available literature on kinetic studies of PCCR with respect to methanol.

Several steps are required in the modeling of PCCR. The first step is to develop the lowest scale or quantum level models to develop DFT and estimate the surface area of the photocatalytic material. Based on this, the reaction rate constants can be estimated from the experiments and the surface reaction network. In the next step, a kMC model is used to evaluate the reaction kinetics using experimental data for validation and comparative studies. Finally, simulations are performed using CFD to optimize the reactors at an industrial or large scale, and the kinetic model can be scaled down. Finally, the results of the perfect kinetic model are used for reactor design and optimization of reaction/operating conditions. For the commercialization of PCCR methanol technology, we need to understand the key points to overcome obstacles, such as (i) the behavior of the reaction mechanism, (ii) the driving force for PCCR, and (iii) how to achieve PCCR efficiency by changing the parameters. Therefore, the kinetic study is important for the solid foundation of CO_2_ photoreduction technology and its development as it plays an important role.

### 3.1 Atomic Scale Modeling

**Figure 16:**
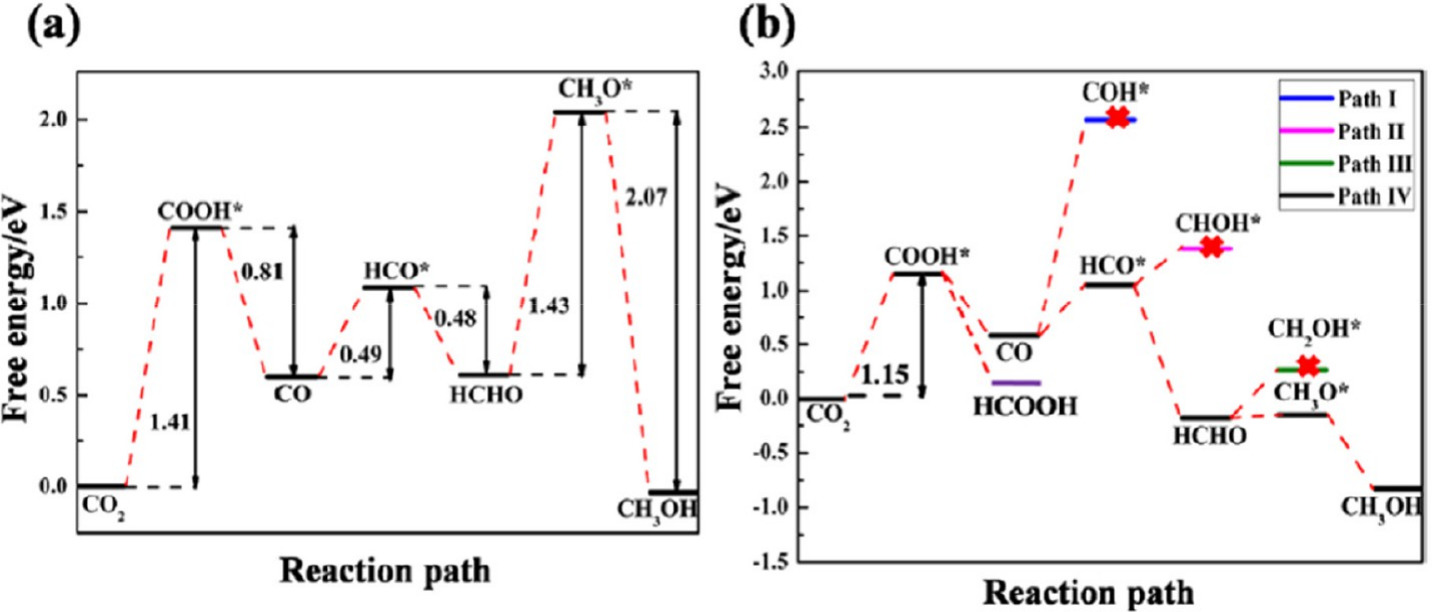
Calculated free energy diagrams to the reaction paths followed by the CO_2_ conversion on (a) g-C_3_N_4_ and (b) S-doped g-C_3_N_4_.^167^ Reproduced with permission from ref (167). Copyright 2018 American Chemical Society.

In atomic level modeling, DFT is the most common and widely used technique. This study is based mainly on quantum computations because they have a favorable cost/performance ratio. Therefore, DFT studies have become very important for several decades to understand the thermodynamics and kinetics of PCCR mechanisms without extended parameters. The literature on selective DFT studies on PCCR to methanol is not overly abundant. In 2018, Wang et al.^167^ investigated sulfur-doped carbon nitride as a photocatalyst for PCCR to methanol using the DFT method, i.e., the DMol^3^ code. The energy profile diagrams for various intermediates formed in the reaction over g-C_3_N_4_ and sulfur-doped g-C_3_N_4_ materials are shown in Figure 16a,b, respectively. From the energy profile diagram, it can be seen that the change in free energy for all the intermediates formed is relatively smaller for the sulfur-doped carbonitride than for the carbonitride. Therefore, the PCCR process is simplified to methanol.

**Figure 17:**
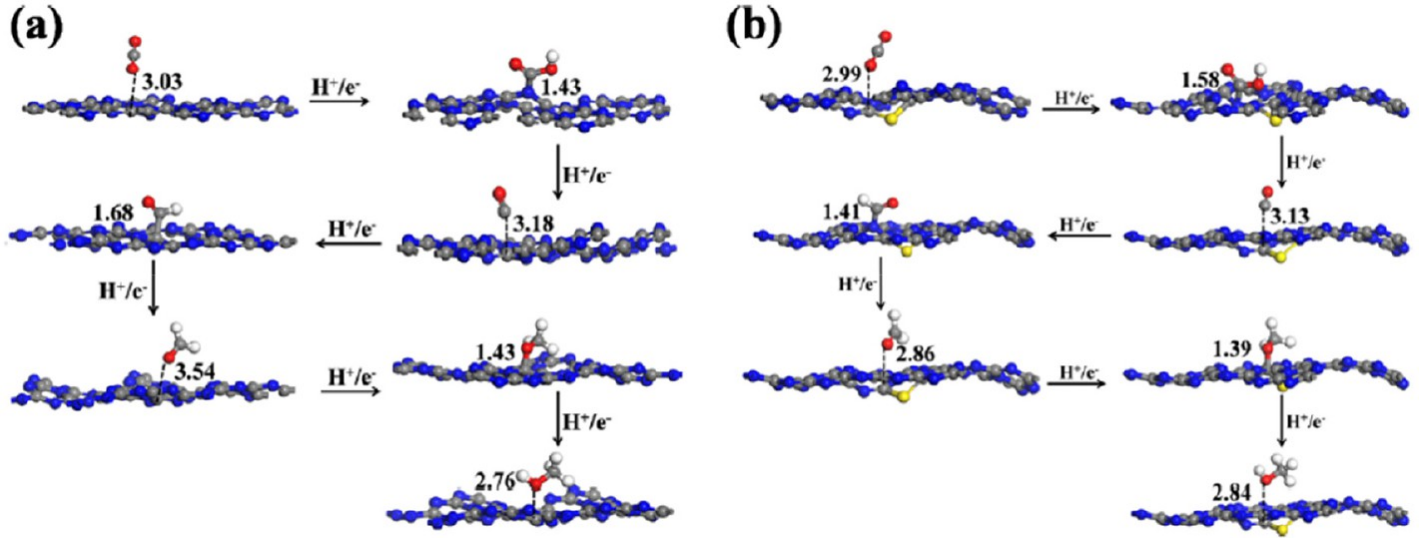
Calculated structures corresponding to the optimal reaction path followed by the CO_2_ conversion on (a) g-C_3_N_4_ and (b) S-doped g-C_3_N_4_. Selected distances are shown in angstrom. Chemisorbed (bound) species are indicated by full bonds, whereas physisorbed species are indicated by dashed bonds. The gray, blue, and yellow balls represent C, N, and S atoms, respectively.^167^ Reproduced with permission from ref (167). Copyright 2018 American Chemical Society.

The authors also calculated the distance between the reacting molecule (reactant, intermediate, and product) and the catalyst surface for g-C_3_N_4_ and sulfur-doped g-C_3_N_4_, and the corresponding interactions are shown in Figure 17a,b. It can be seen that the total energy barrier for PCCR is lower at the surface of the sulfur-doped catalyst, indicating that the relative reaction rate is higher than that of the undoped catalyst. Finally, it can be concluded from the modeling methods that the S-doped catalyst has high PCCR activity.

**Figure 18:**
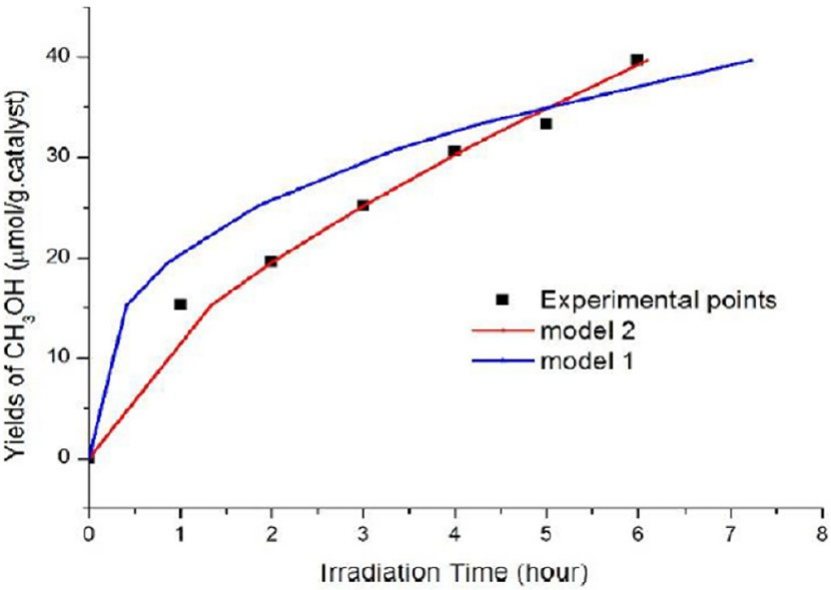
Comparison of model fitting with the experimental data for the formation of CH_3_OH on the CeO_2_=TiO_2_ catalyst.^168^ Reproduced with permission from ref (168). Copyright 2019 IOP Publishing.

Abdullah et al.^168^ used the Langmuir–Hinshelwood kinetic model to estimate the kinetic factors and identify the possible PCCR mechanism *via* the CeO_2_-TiO_2_ catalyst. A nonlinear regression method (i.e., Levenberg–Marquardt) was used to estimate the kinetic constants using Polymath 6.1 software. The authors matched the experimental data of PCCR to CH_3_OH with the kinetic equations. Figure 18 shows the proposed model data along with the experimental results.

This atomic level modeling study is used to understand the basic concepts such as the identification of reaction pathways and intermediates using DFT calculations. The more progressive data of the interaction between the photocatalyst surface and the protons can be investigated by further kinetic studies and are referred to as meso level modeling.

### 3.2 Meso Scale Modeling

DFT studies have some limitations in providing complete information about the reaction mechanism and deeper surface interactions with different parameters. This can be overcome by microkinetic modeling or kMC simulations. The main advantages of kMC are (i) the possibility to analyze more mechanisms (in a probabilistic way), (ii) studies on long-term effects, catalyst saturation and deactivation, (iii) the possibility to obtain more information on the effects of reaction parameters on catalytic activity, and (iv) the kMC results being useful for further studies on industrial optimization CFD simulations. In photocatalytic processes, the Monte Carlo method can be used to solve the RTE by simulating the trajectory of photons in a medium, including absorption, scattering, and reflection. These kMC results are useful to extend and execute a complete mean-field microkinetic system using the first principle for heterogeneous laminar reaction flows in CFD.

### 3.3 Macroscale Modeling

For large structures, simulations are practically only performed with macroscale models. Macro models are used to simulate the entire laminate. Another major problem is that this model is not able to predict the details of the damage processes occurring in the layers. The main objective of macromodels for thermocatalytic CO_2_ reduction is to accurately predict the behavior of the structure and the numerical efficiency of the macromodels. There are few reports in the literature on macroscale modeling of photoreduction associated with CFD. However, the systems studied are very simple ones, such as CO oxidation. Most of the reported methods are based on traditional approaches to thermocatalytic CO_2_ reduction. Simulation modeling data offers valuable solutions in various industries and disciplines by providing clear insights into complex systems.

**Figure 19:**
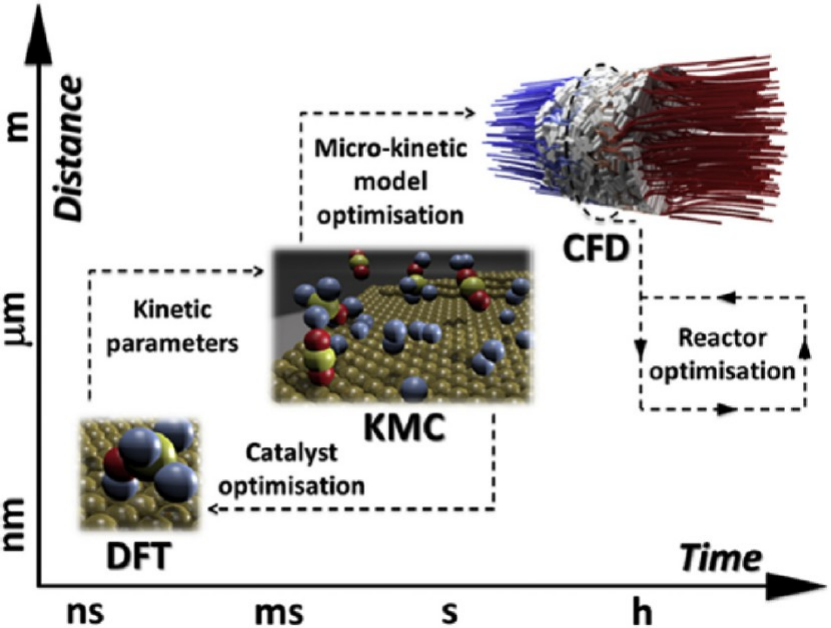
A general approach to multiscale modeling for real unit engineering.^169^ Reproduced with permission from ref (169). Copyright 2020 Elsevier Inc.

Recently, our group^169^ reported on a real-world engineering application using multiscale modeling, which is an important method. However, to date, there has been a lack of development. The authors noted that existing frameworks are generally two-scale models that only link two levels. Further coupling from the small scale to macroscopic transport in a given reactor is still in its infancy, as it is a realistic representation of the microstructure of real catalysts. As shown in Figure 19, it is necessary to obtain a comprehensive description of an operating reactor from the small to the macroscopic scale. In Table 4, we have added the data that summarize and display the modeling parameters at the atomic, meso-, and macroscopic scale.

**Table 4:** Summary and Displays of the Modeling Parameters

Modeling level	Parameter
atomic/quantum-level scale	energetics (thermodynamics), transition states, adsorption, STM images, NMR spectra
molecular dynamics	temporal evolution of the system, mean free path, diffusivity, compressibility
mesoscale	surface diffusion, crystal growth, defect formation, reactions
macroscale	flows, velocities profiles, pressure field pressure drop, computational fluid dynamics (CFD), correlation equations

## 4 Techno-Economic Feasibility of Methanol from Photocatalytic CO_2_ Reduction

Since novel small/laboratory-scale industrial development processes require significant research and development efforts, it is not easy to promote them as innovative technologies or processes. In all the above sections, a high activity/selectivity factor has been considered as the basis for important developments in PCCR to methanol. Nevertheless, there are myriad challenges in conversion from batch to bulk scale, such as desired product selectivity, reaction kinetics, difficult reaction conditions, durability of materials, etc. Any novel technology, such as PCCR to methanol or additional chemical systems, does not spread linearly, and it is stimulating to evaluate how an industrial pathway can be safely achieved at bulk scale. For this, we need a pilot plant investigation, which is an important prerequisite for the commercialization of a process, to determine the actual conditions, parameters, and drawbacks of the experimental data for mass production. In the commercialization phase, we need to verify the market value of our final product, i.e., methanol. This is because methanol is used as an alternative fuel and the price is subject to wide fluctuations.

According to the IEA, global methanol production has increased by 10% annually since 2009. After that, a slight decrease in production was observed, which was 7% and reached 58 million tons in 2012.^170^ According to MMSA calculations, methanol production is 60.6 million tons.^171^ The total global installed capacity was 95.5 million tons in 2012^171^ and 98.3 million tons in the following year 2013, of which 3% was in Europe,^172−174^ mainly in Germany^172^ and Norway.^173^ China is the largest supplier of methanol capacity and consumption in the world, accounting for nearly 50%.^175^ In 2013, it was reported that European plants ensure a capacity factor of almost 82%, while in the US it is 74%.^175^ In Europe, the total production of MeOH was 2.9 million tons in 2013, but the total consumption is estimated to be almost 2.62 times the production, so the rest is covered by imports. According to 2020 records, the typical market value of methanol is around € 270/t, with European spot values around €205/t in Q4 of 2019, after which the Global Impact Coronavirus outbreak (COVID-19) changed the overall picture of methanol prices. European methanol contracts for the first quarter of 2022 were agreed at €495/t ($551/t) at the end of December 2021. Overall, methanol volumes in China are developing significantly, followed by North America, while methanol formation in Europe remains stable.^176^

**Figure 20:**
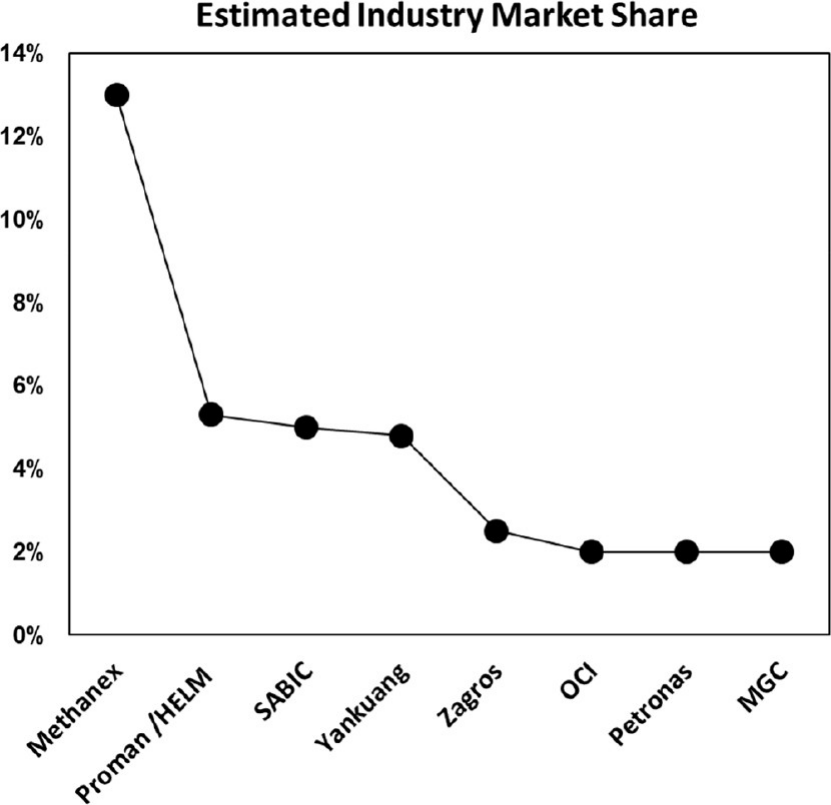
Global estimated industry market share.

Methanex Corporation is an international CH_3_OH pioneer and has a leading market share, a universal production base, and an integrated universal supply chain. This company has an international market share of about 13%, which is twice that of its closest competitor, “HELM -Proman Methanol AG” (see Figure 20). Other companies with a smaller world market share (5–2.5%) include SABIC, Yankuang, Zagros, OCI, Petronas, and MGC. The International Monetary Fund’s (IMF) October 2021 World Economic Outlook states that global demand will grow at about 4% CAGR annually.

The use of methanol as an alternative fuel in public and private facilities has increased worldwide. Initially, the George Olah Plant in Iceland, Europe, established in 2006, produced the renewable fuel using CO_2_ and conducted fleet tests with 100% methanol in special flexible-fuel vehicles from Geely. The Indian government has also decided to promote clean transportation, marine, and power generation applications through fuel cell vehicles. Similarly, the Israeli government is working to reduce its dependence on fuels by using methanol in internal combustion engines developed by Dor Group, the country’s largest company. China, the world’s largest methanol producer and consumer, is also aggressively planning the CH_3_OH fuel market. Some regions in China, with the help of eight ministries and the Ministry of Industry and Information Technology, have issued regulations on the use of methanol vehicles. In Shanxi, Shaanxi, Guizhou, Gansu, and other provinces are accelerating the use of M100 methanol vehicles because they have good reserves and operating experience with CH_3_OH vehicles.

Therefore, it is very important to estimate the production cost before taking the next step, the industrialization of photocatalytic reduction, and this estimation supports the initiation of industrial-scale feasibility. For the techno-economic evaluation of CO_2_ to methanol, the researchers chose “H_2_O splitting” as the model for a pilot-scale demonstration. However, the best techno-economic models showed that light concentration/electricity were the critical aspects for the financial feasibility of PCCR. In 2009, the first demonstration of a pilot plant for photocatalytic splitting of H_2_O was carried out. Liu et al.^177^ presented a review article on the research and development of solar H_2_ production from water at pilot/industrial scales. The system consists of four tubular glass reactors connected in series, each connected to a CPC with an illuminated area of 0.6 m^2^. Under the optimized conditions, hydrogen production is 1.88 L h^–1^, corresponding to an STH of 0.47%. Due to its low capital and operating costs, photocatalytic water splitting has been frequently proposed as a promising method for solar hydrogen production. Therefore, considering such a model is helpful for cost estimation of the PCCR.

**Figure 21:**
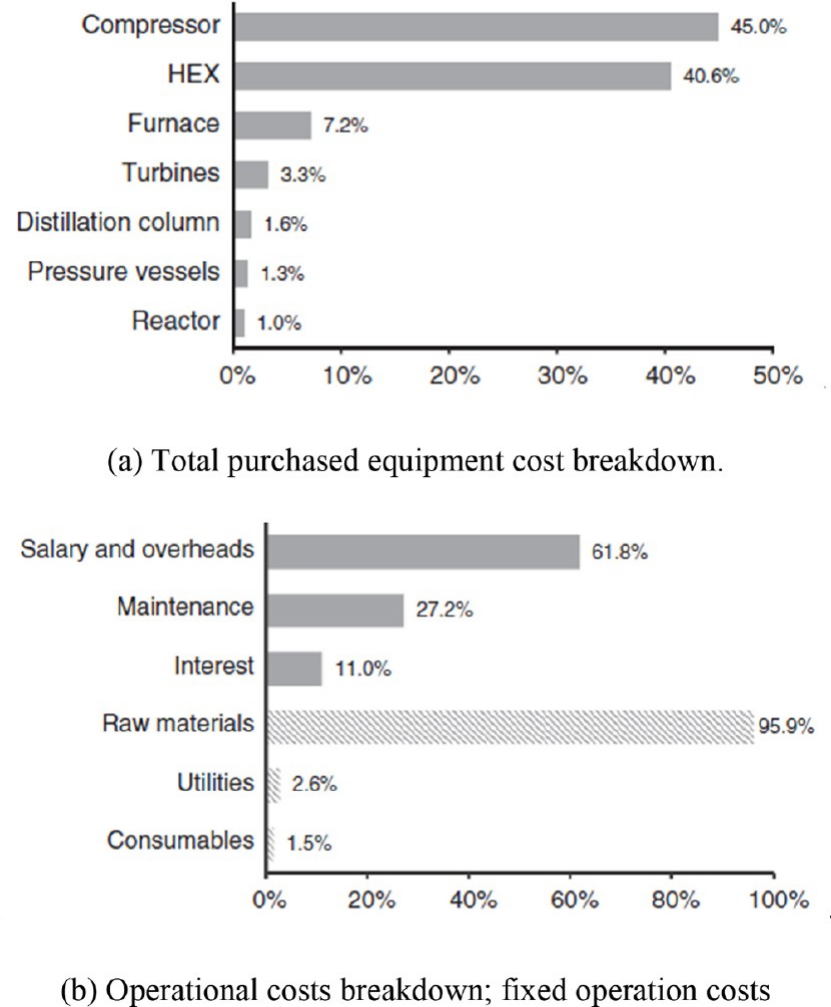
Distribution of the equipment purchasing costs and the operating costs for the MeOH CCU-plant.^178^ Reproduced with permission from ref (178). Copyright 2016 Elsevier Inc.

In 2016, Pérez-Fortes et al.^178^ described the “cost estimation” of a MeOH-CCU plant, which could be helpful in estimating the cost of introducing PCCR in large-scale applications. The authors detailed the equipment and operating costs, which can also be seen in Figure 21. Figure 21a shows that the compression system (45%) accounts for the largest share of the total equipment cost, followed by HEN with a share of 40.6%. The total equipment purchase cost shown is 27 M€. Figure 21b shows the fixed production costs such as operating materials, consumables, maintenance, salaries, etc., with salaries and overheads accounting for the largest share. The variable production costs depend on the market prices of raw materials and the price of H_2_. Therefore, it is necessary to use renewable energy sources (such as wind, solar, and hydro) to achieve a zero-emission energy supply.

Finally, the authors’ assessment of the establishment of the pilot plant is given a formal idea and its feasibility. From the price distribution, it can be seen that the price of electricity has a great impact on the project’s implementation. In general, the cost of electricity varies from country to country, which affects the payback at different locations. Consequently, the main influencing factors are the cost and payback period of CH_3_OH generation and CO_2_ emissions.

For all the conventional methanol synthesis processes mentioned above, the researchers point out that electricity cost may be an important factor for establishing a pilot plant and further synthesis on a large-scale. Therefore, the generation of electricity from renewable energy sources such as solar energy could lead to a lucrative market for photocatalytic reduction of CO_2_.^178^ Photocatalysis directly converts solar energy into chemical energy, which offers a dual advantage of converting CO_2_ into valuable chemicals and storing solar energy. In addition, this overview of photocatalysis could be helpful in using this technology as a potential to solve the current challenges such as energy supply and environmental pollution.

In this review article, we have discussed the critical issues for moving from laboratory to bulk-scale, which will be an important step to lay the foundation for industrial feasibility. The cost of the material will be a critical factor in any business process that is developed. Unfortunately, there are no detailed statistics on price evaluation and market demand to date. This is due to the fact that the entire research community pays special attention to the development of photocatalyst substances and technical aspects. Therefore, we need to pay close attention to the evaluation of the above changes in order to transfer the laboratory scale solutions to the bulk or industrial scale. The evaluation of the world market price and demand is compared for the purpose of commercialization; this is more useful for the development of new scale-up industries and economic evaluation.

## 5 Threats and Opportunities

In this review, we have taken a look at recent research developments in the selective synthesis of PCCR to methanol. From the perspective of photocatalysis, a solution to the problems of greenhouse gases (CO_2_) and increasing energy demand in today’s world has been considered. It is well-known that despite its kinetic and thermodynamic challenges, the PCCR approach has been successfully pursued from the beginning, even though its potential for industrial application was not yet obvious. Under the current strategy of the energy group, our main product, methanol, is also used as a suitable energy storage medium, fuel, hydrocarbon producer, and excellent substitute for petroleum. We have made fundamental distinctions between the conventional CO_2_ thermal conversion reaction, electrocatalysis, and photocatalysis for the core product methanol. We have outlined the main cost-effective and selective processes based on carbonaceous materials and their photocatalytic properties based on industrialization. In addition, the use of state-of-the-art material characterization techniques, kinetic analysis, and computer simulations could raise awareness of this topic.

After a critical review of PCCR to methanol using various photocatalytic materials, it was found that graphene-based photocatalysts achieve high methanol yields. The main advantages of catalysts made of carbonaceous materials are texture and surface properties such as specific surface area, pore volume, pore structure, etc. Metal-based catalysts are also effective as photocatalytic materials. To overcome all these problems, research should focus on developing innovative photoactive, highly stable, and recyclable materials for PCCR. In addition, the solid points of each constituent can be efficiently organized into heterostructure-based materials, which also have enhanced visible light absorption capacity and improved surface and textural properties. From Table 3, it can be seen that the ternary and quaternary photocatalyst materials exhibit high methanol yields due to the incorporation of carbonaceous materials.

Finally, this article provides insights into the design, development of high-efficiency photocatalysts, world market price and demand, operating cost, and selective product methanol from CO_2_ reduction from laboratory to industrial scale. This information will support the establishment of pioneer industries that will take further steps in the near future. With inventions in the field of photocatalyst design, the influence of light and modern technology on simulations/kinetic studies in the photoconversion of CO_2_ may become increasingly important. We expect that new inventions will bring PCCR methanol technology to an industrial level in the near future.

### 5.1 Laboratory-Scale Tests to Large-Scale Feasibility and Assessment Constraints

To date, there is no functioning pilot plant for PCCR to methanol, which has led to a great deal of interest in commercialization research.

1. At the laboratory scale, PCCR to methanol are usually performed at the gram scale (<10 g), but at the large scale, they must be performed at the kilogram scale (>100 kg).
2. Compared to industrial scale, laboratory tests require less energy and light intensity, while industrial tests require a continuous process and low cost, which is a key role/factor. In this regard, carbonaceous material is very interesting in the future because it offers many advantages, such as low cost, high PCCR activity, etc.
3. The lab-scale tests aim at high product selectivity, but the most important factor in this commercial strategy was not only the selectivity of the main product but also the methanol yield.
4. Product separation at laboratory scale is relatively simple, but at large scale it is a major challenge (due to the additional energy required); this principle also needs to be improved.
5. Further research on this topic is needed because, in lab-scale experiments, the price of the work does not accurately reflect the performance, but in large-scale work, the price is significant, which is very specific to PCCR to methanol. Price, demand, and supply in the methanol market are other important issues.
6. For lab-scale testing, many researchers and scientists have indicated a robustness and strength of the catalyst of about 20 to 100 h. However, for industrial or commercial scale, it is important that these values are achieved in a continuous process of 500–10,000 h.

## Abbreviations Used

ACFs: activated carbon fibers
AC: activated carbon
CCS: carbon capture and sequestration
CNTs: carbon nanotubes
CD: carbon dots
CCU: carbon capture and utilization
CB: conduction band
CFD: computational fluid dynamics
CNNA: carbon nitride nanoarrays
CO: carbon monoxide
CN: carbon nitride
CPC: compound parabolic collector
CTAB: cetyltrimethylammonium bromide
DFT: density functional theory
EI: electrochemical impedance
EOR: enhanced oil recovery
EG: expanded graphite
GO: graphene oxide
GP: graphite powder
HEN: heat exchanger network
IEA: International Energy Agency
kMC: kinetic Monte Carlo
LSPR: localized surface plasmon resonance
MOF: metal–organic framework
MWCNTs: multiwalled carbon nanotubes
MMSA: Methanol Market Services Asia
NiO: nickel oxide
NPs: nanoparticles
OMC: ordered mesoporous carbon
RDS: rate-determining step
rG: reduced graphene
rGO: reduced graphene oxide
RTE: radiative transfer equation
S-g-C_3_N_4_: sulfur-doped carbon nitride
STH: solar-to-hydrogen efficiency
TNA: TiO_2_ nanotube array
VB: valence band
